# DL-3-n-butylphthalide for acute ischemic stroke: An updated systematic review and meta-analysis of randomized controlled trials

**DOI:** 10.3389/fphar.2022.963118

**Published:** 2022-09-02

**Authors:** Huan Wang, Kaili Ye, Dan Li, Yuxin Liu, Deren Wang

**Affiliations:** ^1^ Department of Neurology, West China Hospital, Sichuan University, Chengdu, China; ^2^ Department of Psychiatry, Dazhou Central Hospital, Dazhou, China; ^3^ Department of Neurology, Mental and Neurological Disease Research Center, The Third Affiliated Hospital of Sun Yatsen University, Guangzhou, China

**Keywords:** stroke, butylphthalide, efficacy, meta-analysis, systematic review

## Abstract

**Background:** DL -3-n-butylphthalide (NBP) is widely used as a neuroprotective drug in stroke patients in China. A systematic review in 2010 suggested NBP to be safe and effective at promoting neurological recovery, but could not conclude whether it decreased risk of long-term death or disability. Since numerous randomized controlled trials (RCTs) have been conducted on NBP since 2010, we performed an updated systematic review and meta-analysis of safety and efficacy data.

**Method:** We searched electronic databases and reference lists to identify RCTs that compared patients who received NBP or not (including placebo). Methodological quality of RCTs was assessed using the Revised Cochrane Risk of Bias Tool 2.0, and data were meta-analyzed using Review Manager 5.4 software.

**Results:** Fifty-seven RCTs involving 8,747 participants were included. Twenty trials examined NBP as a capsule, 29 as an injection, and 8 as sequential injection-capsule therapy. Meta-analyses showed that NBP treatment was associated with a reduction in composite outcome of death and dependency (risk ratio 0.59, 95% CI 0.42 to 0.83; 260 participants; 2 studies), death (risk ratio 0.32, 95% CI 0.13 to 0.75; 2,287 participants; 10 studies), modified Rankin Scale score (mean difference -0.80, 95% CI -0.88 to -0.72; 568 participants; 4 studies), and an increase in Barthel Index, which assesses the ability to engage in basic activities of daily living (mean difference 11.08, 95% CI 9.10 to 13.05; 2,968 participants; 22 studies). Meta-analyses found that NBP significantly reduced neurological deficit based on National Institute of Health Stroke Scale (mean difference -3.39, 95% CI -3.76 to -3.03; 7.283 participants; 46 studies) and Chinese Stroke Scale (mean difference -4.16, 95% CI -7.60 to -0.73; 543 participants; 4 studies). Of the adverse events reported in 31 trials, elevated transaminase (incidence, 1.39-17.53%), rash (0-1.96%) and gastrointestinal discomfort (1.09-6.15%) were most frequent and no serious adverse events were reported.

**Conclusion:** This update review confirms that NBP can help acute ischemic stroke patients regain the ability to perform activities of daily living, reduce their neurological deficit and short-term death rates. However, the available evidence on whether NBP reduces risk of long-term death or dependence after ischemic stroke remains insufficient.

## Introduction

While the incidence of stroke and its associated mortality has declined in more developed countries, it remains high in China. Each year in that country, approximately 2.5 million individuals suffer a stroke, and 7.5 million live with some form of post-stroke neurological impairment or disability (Wu et al., 2019). The burden of stroke is expected to grow as the population ages (Wang et al., 2017). The only medications using within first few hours recommended by current evidence-based guidelines are recombinant tissue plasminogen activator and antiplatelet therapy (Powers et al., 2019; Kleindorfer et al., 2021). Several neuroprotective drugs may also mitigate stroke-induced injury in experimental studies, yet fail to show robust efficacy in trials (Zhao et al., 2020; Lyden, 2021).

An exception appears to be DL-3-n-butylphthalide (NBP), a drug developed in China that can increase regional cerebral blood flow, reconstruct microcirculation at the ischemic area, inhibit neuronal apoptosis and autophagy, regulate brain energy metabolism, and enhance post-ischemic neuronal recovery (Wang et al., 2013). NBP has also shown anti-inflammatory and anti-oxidant properties (Wang et al., 2013). In 2010, a meta-analysis of 21 randomized controlled trials (RCTs) showed that NBP soft capsules were safe and could improve neurological function recovery after acute ischemic stroke (Wang et al., 2010). However, that meta-analysis was unable to determine whether NBP lowered rates of long-term death or disability after stroke.

Since that meta-analysis, numerous RCTs have examined the safety and efficacy of NBP against acute ischemic stroke, whether as oral, intravenous or sequential intravenous-oral therapy. We therefore performed a systematic review and meta-analysis of the entire evidence base in order to update our understanding of NBP.

## Methods

This systematic review and meta-analysis were performed in accordance with the Preferred Reporting Items for Systematic reviews and Meta-Analyses (PRISMA) statement (Moher et al., 2009).

### Types of studies

We planned to include RCTs in which patients, in addition to conventional treatments, received NBP or not (including placebo) for acute ischemic stroke within 14 days of stroke onset. Only trials involving more than 100 patients that reported the specific method of patient allocation were included. Trials had to be written in English or Chinese. We excluded trials that used quasi-randomization or no randomization, including trials that allocated participants based on alternation, case record number, date of birth, or day of the week.

### Types of participants

Study participants of any age or sex who were diagnosed with acute ischemic stroke according to accepted criteria and were enrolled within 14 days of stroke onset were eligible for inclusion. Accepted diagnostic criteria were those of the fourth or sixth Congress of Chinese Cerebrovascular Diseases (Author Anonymous, 1996; Wang and Wang, 2004), the Chinese Guidelines for Diagnosis and Treatment of Acute Ischemic Stroke from 2010 (Acute Ischemic Stroke Diagnosis and Treatment Guidelines Writing Group Cerebrovascular Diseases Group Neurology Branch of Chinese Medical Association, 2010) , 2014 (Chinese Medical Association Cerebrovascular Diseases Group Neurology Branch of Chinese Medical Association, 2015) or 2018 (Chinese Society of Neurology, 2018), or the World Health Organization (WHO) criteria (Hatano, 1976). Hemorrhagic stroke had to be excluded based on computerized tomography and/or magnetic resonance imaging.

### Types of interventions

Trials could examine NBP of any dosage, treatment duration, or route of administration. The control interventions were placebo or nothing. We included trials involving other drug treatments or other interventions provided they were given to both arms of the trial. Our aim was to investigate two comparisons: (1) NBP vs. placebo, with both arms receiving the same conventional treatment, and (2) NBP vs. no additional treatment or placebo, with both arms receiving the same conventional treatment.

### Types of outcome measures

#### Primary outcome

The primary outcome was the composite outcome of death and dependency after at least 3 months of follow-up. Dependency was defined as dependency on others to perform activities of daily living, which was quantified as a Barthel Index (BI) of 60 or less, or a modified Rankin Scale (mRS) score of 3-5 (Sulter et al., 1999).

#### Secondary outcomes

Secondary outcomes included death from any cause during the scheduled treatment period or follow-up, dependency on others after NBP treatment or at the end of follow-up, and global neurological impairment improvement after NBP treatment or at the end of follow-up, as measured using internationally validated instruments such as the National Institutes of Health Stroke Scale (NIHSS), Canadian Neurological Scale, European Stroke Scale or Scandinavian Stroke Scale. Data were also collected on the following adverse events: nausea, vomiting, allergic reaction, intracranial hemorrhage (symptomatic or asymptomatic), major extracranial hemorrhage, and certain unexplained abnormalities in hepatic, renal, hematological, cardiac or respiratory function.

### Literature search

Through 3 April 2022, we searched the following databases for eligible RCTs: MEDLINE (OVID, 1946 to April 2022), EMBASE (1974 to April 2022), the Cochrane Central Register of Controlled Trials (CENTRAL) (1898 to April 2022), the Chinese National Knowledge Infrastructure (CNKI) (1980 to April 2022), the China Biological Medicine Database (CBM) (1978–2022), the Chinese Science and Technique Journals Database (VIP) (1989–2022), the Chinese Doctoral Dissertations Full-text Database (CDFD), and the Chinese Master’s Theses Full-text Database (CMFD) in CNKI (1999–2022). An example of the search process is shown in Supplementary Table S1.

We also manually searched reference lists of relevant publications and contacted the manufacturer of NBP (CSPC-NBP Pharmaceutical Co., Ltd.) in order to identify additional potential eligible studies.

### Study selection and data extraction

Two reviewers (HW and KY) checked studies for eligibility based initially on the titles and abstracts, and then on the full text. Disagreements were resolved by discussion and, if necessary, the intervention of the senior author (DW).

Data were extracted independently by two reviewers (HW and KY) using a data extraction form. Disagreements were resolved by discussion. Missing data were obtained from the corresponding authors whenever possible.

### Assessment of risk of bias in included studies

Two authors (HW and YL) independently assessed the risk of bias in the included studies, using the revised Cochrane Risk of Bias Tool for Randomized Trials (RoB 2) with the Microsoft Excel template (version of August 2019) (Higgins et al., 2016). We assessed risk of the following types of bias: bias in the randomization process, bias due to deviations from intended interventions, bias due to missing outcome data, bias in the measurement of the outcome, and bias in the selection of the reported result. Risk was categorized as “low”, “some concern”, or “high”. We judged a study to be at high overall risk of bias when risk of bias was high for at least one domain; a study was judged to be at low overall risk of bias when the risk was low for all domains. Discrepant assessments by the two authors were resolved through discussions involving the senior author (DW).

### Meta-analysis

We performed meta-analyses using RevMan 5.4 (The Cochrane Collaboration, 2020). We presented pooled results as risk ratios (RRs) with 95% confidence intervals (CIs) for dichotomous outcomes, and mean differences (MDs) or standardized mean differences (SMDs) and 95% CIs for continuous outcomes. We meta-analyzed data using a fixed-effect model if no substantial statistical heterogeneity was present; otherwise, we used a random-effects statistical model. We assessed heterogeneity using the I^2^ statistic, with a value greater than 50% indicating substantial heterogeneity. We planned to perform subgroup analyses of different forms of NBP (soft capsules, injections, or sequential therapy), if sufficient trials had been available (at least 10 trials per outcome). We planned to perform sensitivity analyses by excluding trials whose overall risk of bias was “high” or “some concern”. Publication bias was assessed by generating a funnel plot for the outcome for which the largest number of trials could be meta-analyzed.

## Results

### Description of studies

**FIGURE 1:**
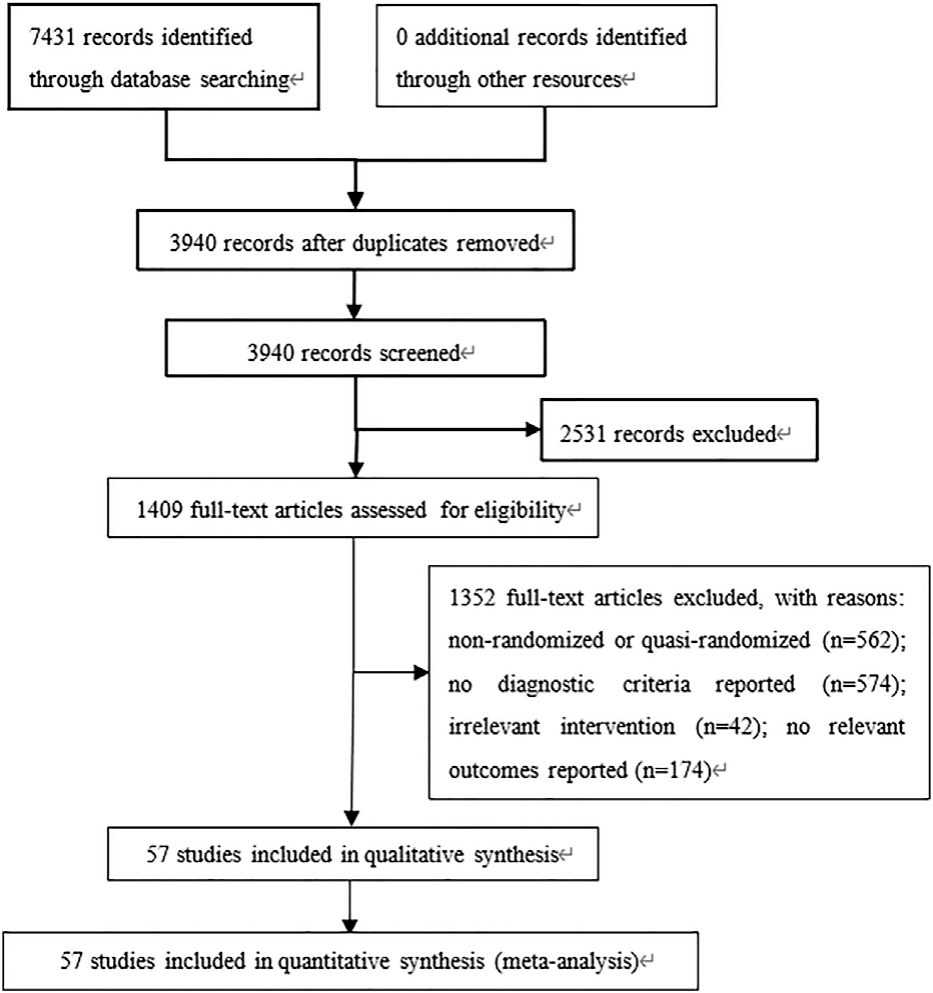
Flow diagram of study inclusion.

A total of 7,431 relevant publications were identified, of which 3,491 were excluded as duplicates (Figure 1). Another 2,531 were excluded on the basis of their titles and abstracts, leaving 1,409 whose full text was reviewed. After excluding 1,352 studies because they did not fulfill the inclusion criteria or because they failed to report the necessary outcome data, we were left with 57 studies involving 8,747 participants (Cui et al., 2005a; Cui et al., 2005b; Wang and Li, 2016; Fu, 2015; Yu, 2018; Liu et al., 2018a; Lv et al., 2018; Lv, 2015; Wu, 2019; Zhou et al., 2015; Chang and Ma, 2018; Chang, 2018; Zhang, 2018; Zhang et al., 2018a; Zhang and Li, 2018; Zhang et al., 2018b; Xu et al., 2006; Li, 2017a; Li et al., 2017; Li, 2017b; Li, 2018; Lin et al., 2018; Xiong and Hong, 2018; Bai, 2019; Qin and Han, 2019; Fu et al., 2017; Dong et al., 2016; Xu et al., 2016; Xu, 2018; Zheng et al., 2016; Chen et al., 2019; Wei et al., 2012; Yan and Ma, 2015; Ma and Xiao, 2018; Gao et al., 2017; Jin, 2019; Pan et al., 2019; Jiang, 2019; Li et al., 2020a; Wang et al., 2020a; Wang, 2020; Zhang et al., 2020; Zhou et al., 2020). All studies were conducted in Chinese hospitals (Supplementary Table S2).

The control arm in three trials received conventional treatment as well as placebo instead of NBP (Cui et al., 2005a; Cui et al., 2005b; Wang and Li, 2016), while the control arm in 54 trials received conventional treatment without placebo or NBP (Fu, 2015; Yu, 2018; Liu et al., 2018a; Lv et al., 2018; Lv, 2015; Wu, 2019; Zhou et al., 2015; Chang and Ma, 2018; Chang, 2018; Zhang, 2018; Zhang et al., 2018a; Zhang and Li, 2018; Zhang et al., 2018b; Xu et al., 2006; Li, 2017a; Li et al., 2017; Li, 2017b; Li, 2018; Lin et al., 2018; Xiong and Hong, 2018; Bai, 2019; Qin and Han, 2019; Fu et al., 2017; Dong et al., 2016; Xu et al., 2016; Xu, 2018; Zheng et al., 2016; Chen et al., 2019; Wei et al., 2012; Yan and Ma, 2015; Ma and Xiao, 2018; Gao et al., 2017; Jin, 2019; Pan et al., 2019; Jiang, 2019; Li et al., 2020a; Wang et al., 2020a; Wang, 2020; Zhang et al., 2020; Zhou et al., 2020; Chen and Qian, 2021; Li, 2021; Liu et al., 2021a). The intervention arm in all trials received NBP and conventional treatment, which included recombinant tissue plasminogen activator, urinary kallidinogenase, anticoagulants, antiplatelets, statins, traditional Chinese medicine, neuroprotective drugs, or early rehabilitation. The NBP formulation was a soft capsule in 20 trials (Cui et al., 2005a; Cui et al., 2005b; Xu et al., 2006; Wei et al., 2012; Lv, 2015; Wang and Li, 2016; Li, 2017b; Fu et al., 2017; Li, 2018; Lin et al., 2018; Xiong and Hong, 2018; Xu, 2018; Jiang, 2019; Li et al., 2020a; Zhang et al., 2020; Zhu et al., 2021a; Zhu et al., 2021b; Chen et al., 2021; Chen and Qian, 2021; Si et al., 2022), an injection in 29 trials (Fu, 2015; Zhou et al., 2015; Dong et al., 2016; Xu et al., 2016; Li, 2017a; Gao et al., 2017; Li et al., 2017; Zhang et al., 2018b; Chang, 2018; Chang and Ma, 2018; Lv et al., 2018; Ma and Xiao, 2018; Yu, 2018; Zhang, 2018; Zhang and Li, 2018; Bai, 2019; Chen et al., 2019; Jin, 2019; Wu, 2019; Wang et al., 2020a; Wang, 2020; Liu et al., 2021a; Li, 2021; Pang et al., 2021; Yang et al., 2021; Ye et al., 2021; Zhang et al., 2021; Wu, 2022; Zhu, 2022), or sequential intravenous-capsule treatment in the remaining eight trials (Yan and Ma, 2015; Zheng et al., 2016; Liu et al., 2018a; Zhang et al., 2018a; Pan et al., 2019; Qin and Han, 2019; Wang et al., 2020b; Zhou et al., 2020). Twenty trials used NBP soft capsules at a dose of 200 mg tid (Wei et al., 2012; Lv, 2015; Li, 2017b; Fu et al., 2017; Li, 2018; Lin et al., 2018; Xiong and Hong, 2018; Xu, 2018; Jiang, 2019; Li et al., 2020a; Zhu et al., 2021a; Zhu et al., 2021b; Chen et al., 2021; Chen and Qian, 2021; Si et al., 2022) or 200 mg qid (Cui et al., 2005a; Cui et al., 2005b; Xu et al., 2006; Wang and Li, 2016; Zhang et al., 2020), and treatment lasted 2 weeks (Lv, 2015; Wang and Li, 2016; Fu et al., 2017; Li, 2018; Lin et al., 2018; Xiong and Hong, 2018; Jiang, 2019; Li et al., 2020a; Zhu et al., 2021b; Chen and Qian, 2021; Si et al., 2022), 3 weeks (20-21 days) (Cui et al., 2005a; Cui et al., 2005b; Xu et al., 2006; Wei et al., 2012; Li, 2017b; Zhang et al., 2020), 1 month (Zhu et al., 2021a) or 3 months (Xu, 2018; Chen et al., 2021). Twenty-nine trials used NBP injections of 100 ml bid (Fu, 2015; Zhou et al., 2015; Dong et al., 2016; Xu et al., 2016; Li, 2017a; Gao et al., 2017; Li et al., 2017; Zhang et al., 2018b; Chang, 2018; Chang and Ma, 2018; Lv et al., 2018; Ma and Xiao, 2018; Yu, 2018; Zhang, 2018; Zhang and Li, 2018; Bai, 2019; Chen et al., 2019; Jin, 2019; Wu, 2019; Wang et al., 2020a; Wang, 2020; Liu et al., 2021a; Pang et al., 2021; Yang et al., 2021; Ye et al., 2021; Zhang et al., 2021; Wu, 2022; Zhu, 2022) or 100 ml qd (Li, 2021), with each 100-ml injection containing 25 mg of NBP and 0.9 g of sodium chloride; such treatment lasted 2 weeks (Fu, 2015; Dong et al., 2016; Xu et al., 2016; Li, 2017a; Gao et al., 2017; Li et al., 2017; Zhang et al., 2018b; Chang, 2018; Chang and Ma, 2018; Lv et al., 2018; Ma and Xiao, 2018; Yu, 2018; Zhang, 2018; Zhang and Li, 2018; Bai, 2019; Chen et al., 2019; Wu, 2019; Wang et al., 2020a; Wang, 2020; Liu et al., 2021a; Li, 2021; Pang et al., 2021; Yang et al., 2021; Ye et al., 2021; Zhang et al., 2021; Zhu, 2022), 3 weeks (Zhou et al., 2015) or 1 month (Jin, 2019; Wu, 2022). In the eight trials using sequential NBP therapy (Yan and Ma, 2015; Zheng et al., 2016; Liu et al., 2018a; Zhang et al., 2018a; Pan et al., 2019; Qin and Han, 2019; Wang et al., 2020b; Zhou et al., 2020), an injection of 100 ml bid was given for the first seven (Yan and Ma, 2015), 10 (Pan et al., 2019; Wang et al., 2020b) or 14 days (Zheng et al., 2016; Liu et al., 2018a; Zhang et al., 2018a; Qin and Han, 2019; Zhou et al., 2020), followed by soft capsules at 100 mg tid (Qin and Han, 2019; Wang et al., 2020b) or 200 mg tid (Yan and Ma, 2015; Zheng et al., 2016; Liu et al., 2018a; Zhang et al., 2018a; Pan et al., 2019; Zhou et al., 2020) for the next 7 days (Yan and Ma, 2015), 2 weeks (Zhou et al., 2020), 1 month (Zhang et al., 2018a; Pan et al., 2019; Wang et al., 2020b) or 3 months (Zheng et al., 2016; Liu et al., 2018a; Qin and Han, 2019).

Two trials reported the composite outcome of death and dependency at 3 months of follow-up (Pan et al., 2019; Zhou et al., 2020). Ten trials reported the number of deaths after treatment for 14 days (Dong et al., 2016; Wang and Li, 2016; Gao et al., 2017; Zhang et al., 2018b; Chang and Ma, 2018; Pang et al., 2021) or 21 days (Wei et al., 2012; Zhou et al., 2015), or at the end of follow-up lasting 3 months (Wang et al., 2020b) or 5 months (Yang et al., 2021). Thirty-two trials assessed dependency, among which six trials used mRS score after NBP treatment (Wang, 2020; Zhang et al., 2020; Yang et al., 2021; Wu, 2022), or at 3 months of follow-up (Wang et al., 2020b; Ye et al., 2021). Twenty-three trials used the BI (Fu, 2015; Zhou et al., 2015; Zheng et al., 2016; Li, 2017a; Fu et al., 2017; Gao et al., 2017; Li et al., 2017; Liu et al., 2018a; Zhang et al., 2018a; Lin et al., 2018; Lv et al., 2018; Ma and Xiao, 2018; Xu, 2018; Zhang and Li, 2018; Qin and Han, 2019; Chen et al., 2021; Chen and Qian, 2021; Yang et al., 2021; Ye et al., 2021; Zhang et al., 2021; Si et al., 2022; Wu, 2022; Zhu, 2022) after NBP treatment (Zhou et al., 2015; Zheng et al., 2016; Fu et al., 2017; Gao et al., 2017; Liu et al., 2018a; Zhang et al., 2018a; Lin et al., 2018; Xu, 2018; Zhang and Li, 2018; Qin and Han, 2019; Chen et al., 2021; Chen and Qian, 2021; Ye et al., 2021; Zhang et al., 2021; Si et al., 2022; Wu, 2022; Zhu, 2022) at follow-up of either 1 month (Fu, 2015) or 3 months (Li, 2017a; Li et al., 2017; Lv et al., 2018; Ma and Xiao, 2018; Yang et al., 2021). Six trials did not clearly describe the numerical scoring system that they used (Lv, 2015; Zhang et al., 2018b; Chang, 2018; Xiong and Hong, 2018; Chen et al., 2019; Li, 2021). Three of 32 trials used both mRS and the BI (Yang et al., 2021; Ye et al., 2021; Wu, 2022). Only one trial reported dependency rate (Ye et al., 2021), and the remaining trials reported scores as means and standard deviations (SDs).

All but two trials (Jiang, 2019; Yang et al., 2021) reported global neurological impairment improvement after treatment or at the end of follow-up; 48 trials reported changes in NIHSS score (Wang and Li, 2016; Fu, 2015; Yu, 2018; Liu et al., 2018a; Lv et al., 2018; Lv, 2015; Wu, 2019; Zhou et al., 2015; Chang and Ma, 2018; Chang, 2018; Zhang, 2018; Zhang et al., 2018a; Zhang and Li, 2018; Zhang et al., 2018b; Li, 2017a; Li et al., 2017; Li, 2017b; Lin et al., 2018; Xiong and Hong, 2018; Bai, 2019; Qin and Han, 2019; Dong et al., 2016; Xu et al., 2016; Xu, 2018; Zheng et al., 2016; Chen et al., 2019; Yan and Ma, 2015; Ma and Xiao, 2018; Gao et al., 2017; Jin, 2019; Pan et al., 2019; Li et al., 2020a; Wang et al., 2020a; Wang, 2020; Zhang et al., 2020; Zhou et al., 2020; Chen and Qian, 2021; Li, 2021; Liu et al., 2021a; Pang et al., 2021; Ye et al., 2021; Zhu et al., 2021a; Zhu et al., 2021b) after treatment (Lv, 2015; Yan and Ma, 2015; Zhou et al., 2015; Dong et al., 2016; Wang and Li, 2016; Xu et al., 2016; Zheng et al., 2016; Li, 2017a; Li, 2017b; Gao et al., 2017; Li et al., 2017; Liu et al., 2018a; Zhang et al., 2018a; Zhang et al., 2018b; Chang and Ma, 2018; Lin et al., 2018; Lv et al., 2018; Xiong and Hong, 2018; Xu, 2018; Yu, 2018; Zhang, 2018; Zhang and Li, 2018; Chen et al., 2019; Jin, 2019; Qin and Han, 2019; Wu, 2019; Li et al., 2020a; Wang et al., 2020a; Wang, 2020; Zhang et al., 2020; Liu et al., 2021a; Zhu et al., 2021a; Zhu et al., 2021b; Chen et al., 2021; Chen and Qian, 2021; Li, 2021; Pang et al., 2021; Ye et al., 2021; Si et al., 2022; Wu, 2022; Zhu, 2022) or during follow-up of up to 1 month (Fu, 2015; Chang, 2018; Bai, 2019) or 3 months (Ma and Xiao, 2018; Pan et al., 2019; Wang et al., 2020b; Zhou et al., 2020). Another four trials reported changes in Chinese Stroke Scale (CSS) score (Cui et al., 2005a; Cui et al., 2005b; Wei et al., 2012; Li, 2018) at the end of treatment lasting 14 days (Li, 2018) or 21 days (Cui et al., 2005a; Cui et al., 2005b; Wei et al., 2012), while two trials reported changes in the modified Edinburgh-Scandinavia Stroke Scale (MESSS) (Zhang et al., 2021) or Cerebrovascular Disease Rehabilitation Medical Program and Assessment Criteria Scale score (Xu et al., 2006). One trial reported changes in an unidentified scoring system at the end of 14-days treatment (Fu et al., 2017).

Of the 57 trials, 31 reported that adverse events occurred (Cui et al., 2005a; Cui et al., 2005b; Xu et al., 2006; Zhou et al., 2015; Wang and Li, 2016; Zheng et al., 2016; Li, 2017a; Gao et al., 2017; Zhang et al., 2018a; Chang, 2018; Ma and Xiao, 2018; Xiong and Hong, 2018; Yu, 2018; Zhang and Li, 2018; Bai, 2019; Jiang, 2019; Jin, 2019; Qin and Han, 2019; Wu, 2019; Li et al., 2020a; Wang, 2020; Zhang et al., 2020; Zhou et al., 2020; Liu et al., 2021a; Li, 2021; Pang et al., 2021; Ye et al., 2021; Zhang et al., 2021; Si et al., 2022; Wu, 2022; Zhu, 2022), 10 reported that no adverse events occurred (Fu, 2015; Yan and Ma, 2015; Dong et al., 2016; Fu et al., 2017; Li et al., 2017; Li, 2018; Zhang, 2018; Pan et al., 2019; Wang et al., 2020a; Yang et al., 2021), and the remaining 16 did not mention whether adverse events occurred or not (Wei et al., 2012; Lv, 2015; Xu et al., 2016; Li, 2017b; Liu et al., 2018a; Zhang et al., 2018b; Chang and Ma, 2018; Lin et al., 2018; Lv et al., 2018; Xu, 2018; Chen et al., 2019; Wang et al., 2020b; Zhu et al., 2021a; Zhu et al., 2021b; Chen et al., 2021; Chen and Qian, 2021).

### Risk of bias in included studies

**FIGURE 2:**
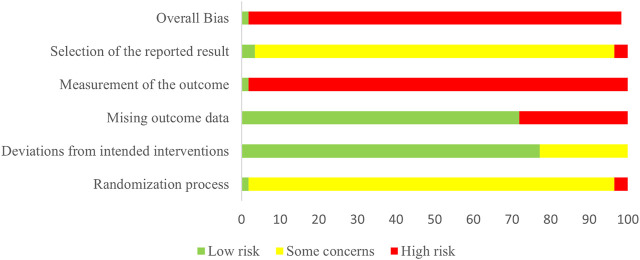
Risk of bias in the included trials.

Risk of bias for all the included studies is assessed in Figure 2 and Supplementary Figure S1. In the domain of randomization, one trial (Cui et al., 2005b) was evaluated at low risk of bias, 54 at some concern (Cui et al., 2005a; Wang and Li, 2016; Fu, 2015; Yu, 2018; Lv et al., 2018; Lv, 2015; Wu, 2019; Zhou et al., 2015; Chang and Ma, 2018; Chang, 2018; Zhang, 2018; Zhang et al., 2018a; Zhang and Li, 2018; Zhang et al., 2018b; Xu et al., 2006; Li et al., 2017; Li, 2017b; Li, 2018; Lin et al., 2018; Xiong and Hong, 2018; Bai, 2019; Qin and Han, 2019; Fu et al., 2017; Dong et al., 2016; Xu et al., 2016; Xu, 2018; Zheng et al., 2016; Chen et al., 2019; Wei et al., 2012; Yan and Ma, 2015; Ma and Xiao, 2018; Gao et al., 2017; Jin, 2019; Pan et al., 2019; Jiang, 2019; Li et al., 2020a; Wang et al., 2020a; Wang, 2020; Zhang et al., 2020; Zhou et al., 2020; Chen and Qian, 2021; Li, 2021; Liu et al., 2021a), and two at high risk of bias (Li, 2017a; Liu et al., 2018a). In the domain of deviations from intended interventions, 44 trials (Cui et al., 2005a; Cui et al., 2005b; Wang and Li, 2016; Fu, 2015; Lv, 2015; Zhou et al., 2015; Chang and Ma, 2018; Chang, 2018; Zhang, 2018; Zhang et al., 2018a; Zhang and Li, 2018; Zhang et al., 2018b; Xu et al., 2006; Li, 2017a; Li et al., 2017; Li, 2017b; Lin et al., 2018; Bai, 2019; Qin and Han, 2019; Fu et al., 2017; Dong et al., 2016; Xu et al., 2016; Xu, 2018; Wei et al., 2012; Yan and Ma, 2015; Ma and Xiao, 2018; Gao et al., 2017; Jin, 2019; Pan et al., 2019; Jiang, 2019; Wang et al., 2020a; Wang, 2020; Zhou et al., 2020; Chen and Qian, 2021; Li, 2021; Pang et al., 2021; Yang et al., 2021; Ye et al., 2021; Zhang et al., 2021; Zhu et al., 2021a; Zhu et al., 2021b; Chen et al., 2021; Si et al., 2022) were evaluated at low risk of bias, and 13 at some concern (Zheng et al., 2016; Liu et al., 2018a; Li, 2018; Lv et al., 2018; Xiong and Hong, 2018; Yu, 2018; Chen et al., 2019; Wu, 2019; Li et al., 2020a; Wang et al., 2020b; Zhang et al., 2020; Liu et al., 2021a; Zhu, 2022). In the domain of missing outcome data, 41 trials (Cui et al., 2005b; Xu et al., 2006; Wei et al., 2012; Fu, 2015; Lv, 2015; Yan and Ma, 2015; Zhou et al., 2015; Dong et al., 2016; Wang and Li, 2016; Xu et al., 2016; Li, 2017a; Li, 2017b; Fu et al., 2017; Gao et al., 2017; Li et al., 2017; Zhang et al., 2018a; Zhang et al., 2018b; Chang, 2018; Chang and Ma, 2018; Lin et al., 2018; Ma and Xiao, 2018; Xu, 2018; Zhang, 2018; Zhang and Li, 2018; Bai, 2019; Jiang, 2019; Jin, 2019; Qin and Han, 2019; Wang et al., 2020a; Wang, 2020; Zhou et al., 2020; Zhu et al., 2021a; Zhu et al., 2021b; Chen et al., 2021; Chen and Qian, 2021; Li, 2021; Pang et al., 2021; Yang et al., 2021; Zhang et al., 2021; Si et al., 2022; Wu, 2022) were evaluated at low risk of bias, and the remaining 16 (Cui et al., 2005a; Zheng et al., 2016; Liu et al., 2018a; Li, 2018; Lv et al., 2018; Xiong and Hong, 2018; Yu, 2018; Chen et al., 2019; Pan et al., 2019; Wu, 2019; Li et al., 2020a; Wang et al., 2020b; Zhang et al., 2020; Liu et al., 2021a; Ye et al., 2021; Zhu, 2022) at high risk of bias. In the domain of outcome measurement, one trial (Cui et al., 2005b) was evaluated at low risk of bias and 56 (Cui et al., 2005a; Wang and Li, 2016; Fu, 2015; Yu, 2018; Liu et al., 2018a; Lv et al., 2018; Lv, 2015; Wu, 2019; Zhou et al., 2015; Chang and Ma, 2018; Chang, 2018; Zhang, 2018; Zhang et al., 2018a; Zhang and Li, 2018; Zhang et al., 2018b; Xu et al., 2006; Li, 2017a; Li et al., 2017; Li, 2017b; Li, 2018; Lin et al., 2018; Xiong and Hong, 2018; Bai, 2019; Qin and Han, 2019; Fu et al., 2017; Dong et al., 2016; Xu et al., 2016; Xu, 2018; Zheng et al., 2016; Chen et al., 2019; Wei et al., 2012; Yan and Ma, 2015; Ma and Xiao, 2018; Gao et al., 2017; Jin, 2019; Pan et al., 2019; Jiang, 2019; Li et al., 2020a; Wang et al., 2020a; Wang, 2020; Zhang et al., 2020; Zhou et al., 2020; Chen and Qian, 2021) at high risk of bias. In the domain of selection of the reported result, two trials (Cui et al., 2005a; Cui et al., 2005b) were evaluated at low risk of bias, 53 (Xu et al., 2006; Wei et al., 2012; Fu, 2015; Yan and Ma, 2015; Zhou et al., 2015; Dong et al., 2016; Wang and Li, 2016; Xu et al., 2016; Zheng et al., 2016; Li, 2017a; Li, 2017b; Fu et al., 2017; Gao et al., 2017; Li et al., 2017; Liu et al., 2018a; Zhang et al., 2018a; Zhang et al., 2018b; Chang, 2018; Chang and Ma, 2018; Li, 2018; Lin et al., 2018; Lv et al., 2018; Ma and Xiao, 2018; Xiong and Hong, 2018; Xu, 2018; Yu, 2018; Zhang, 2018; Zhang and Li, 2018; Bai, 2019; Chen et al., 2019; Jiang, 2019; Jin, 2019; Pan et al., 2019; Qin and Han, 2019; Wu, 2019; Wang et al., 2020a; Wang et al., 2020b; Wang, 2020; Zhang et al., 2020; Zhou et al., 2020; Liu et al., 2021a; Zhu et al., 2021a; Zhu et al., 2021b; Chen et al., 2021; Chen and Qian, 2021; Li, 2021; Pang et al., 2021; Yang et al., 2021; Ye et al., 2021; Zhang et al., 2021; Si et al., 2022; Wu, 2022; Zhu, 2022) at some concern, and two (Lv, 2015; Li et al., 2020a) at high risk of bias. One trial (Cui et al., 2005b) was categorized as being at low overall risk of bias, and the remaining 56 (Cui et al., 2005a; Wang and Li, 2016; Fu, 2015; Yu, 2018; Liu et al., 2018a; Lv et al., 2018; Lv, 2015; Wu, 2019; Zhou et al., 2015; Chang and Ma, 2018; Chang, 2018; Zhang, 2018; Zhang et al., 2018a; Zhang and Li, 2018; Zhang et al., 2018b; Xu et al., 2006; Li, 2017a; Li et al., 2017; Li, 2017b; Li, 2018; Lin et al., 2018; Xiong and Hong, 2018; Bai, 2019; Qin and Han, 2019; Fu et al., 2017; Dong et al., 2016; Xu et al., 2016; Xu, 2018; Zheng et al., 2016; Chen et al., 2019; Wei et al., 2012; Yan and Ma, 2015; Ma and Xiao, 2018; Gao et al., 2017; Jin, 2019; Pan et al., 2019; Jiang, 2019; Li et al., 2020a; Wang et al., 2020a; Wang, 2020; Zhang et al., 2020; Zhou et al., 2020; Chen and Qian, 2021) as being at high overall risk.

### Composite outcome of death and dependency

**FIGURE 3:**
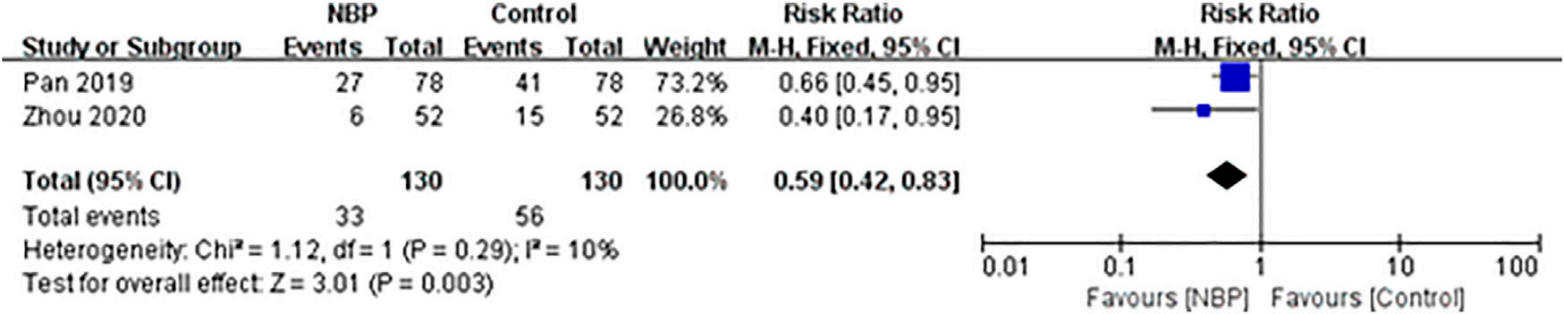
Meta-analysis of the composite outcome of death and dependency at 3-months follow-up.

Two trials involving 264 participants, of whom 4 (1.52%) were lost to follow-up, reported the composite outcome of death and dependency at 3 months of follow-up (Pan et al., 2019; Zhou et al., 2020). The incidence of death and dependency was 25.38% (33/130) in the intervention group and 43.08% (56/130) in the control group, and fixed-effect meta-analysis showed evidence for a protective effect of NBP against the composite outcome of death and dependency (RR 0.59, 95% CI 0.42 to 0.83; participants = 260; studies = 2; I^2^ = 10%; Figure 3).

### Death

Ten trials reported the number of deaths during the treatment period or at follow-up. One trial (Zhang et al., 2018b) reported one death in the intervention group (1/73, 1.37%) and four deaths in the control group (4/73, 5.48%) during 14-days treatment. One trial (Pang et al., 2021) reported one death in the intervention group (1/53, 1.89%) and seven deaths in the control group (7/52, 13.46%) at the end of 14-days treatment. One trial (Dong et al., 2016) reported only one death in the control group during 14-days treatment (1/86, 1.16%). One trial (Wei et al., 2012) reported one death in each group at the end of 21-days treatment (both 1/55, 1.82%). One trial (Wang et al., 2020b) reported two deaths in the intervention group (2/89, 2.25%) and three deaths in the control group (3/89, 3.37%) during 3-months follow-up. One trial (Yang et al., 2021) reported one death in the intervention group (1/71, 2.44%) and four deaths in the control group (4/71, 5.63%) during 5-months follow-up. The other four trials reported no deaths during 14-days treatment (Wang and Li, 2016; Gao et al., 2017; Chang and Ma, 2018) or 21-days treatment (Zhou et al., 2015).

**FIGURE 4:**
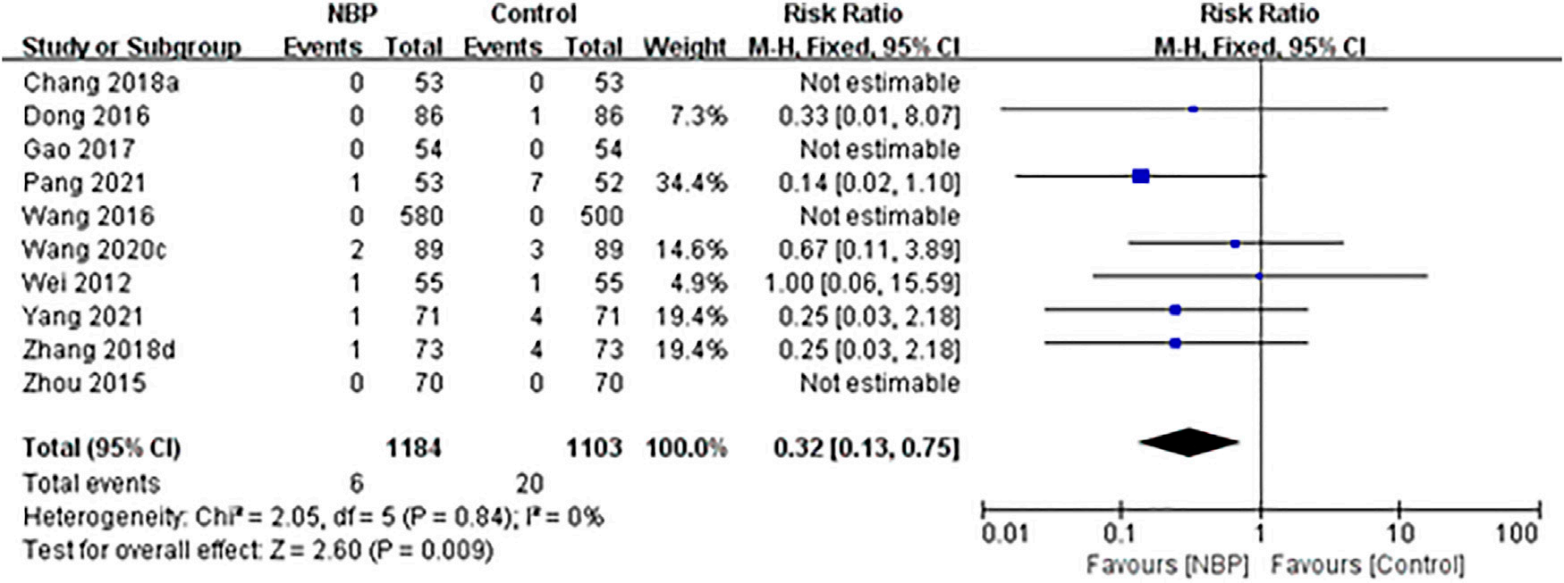
Meta-analysis of death during the treatment period or follow-up.

Across all 10 trials, the incidence of death was 0.51% (6/1,184) in the intervention group and 1.81% (20/1,103) in the control group, and fixed-effect meta-analysis showed that NBP treatment was associated with a significant reduction in death during the treatment period or at follow-up (RR 0.32, 95% CI 0.13 to 0.75; participants = 2,287; studies = 10; I^2^ = 0%; Figure 4).

### Dependency

#### mRS

Six trials used the mRS score to access the level of functional independence after NBP treatment (Wang, 2020; Zhang et al., 2020; Yang et al., 2021; Wu, 2022) or at 3-months follow-up (Wang et al., 2020b; Ye et al., 2021). One did not report the specific scores of mRS (Wang et al., 2020b). One trial (Ye et al., 2021) involving 204 participants, of whom 10 (4.90%) were lost to 3-months follow-up, reported that the dependency rate was 21.43% (21/98) in the intervention group and 36.46% (35/96) in the control group.

**FIGURE 5:**
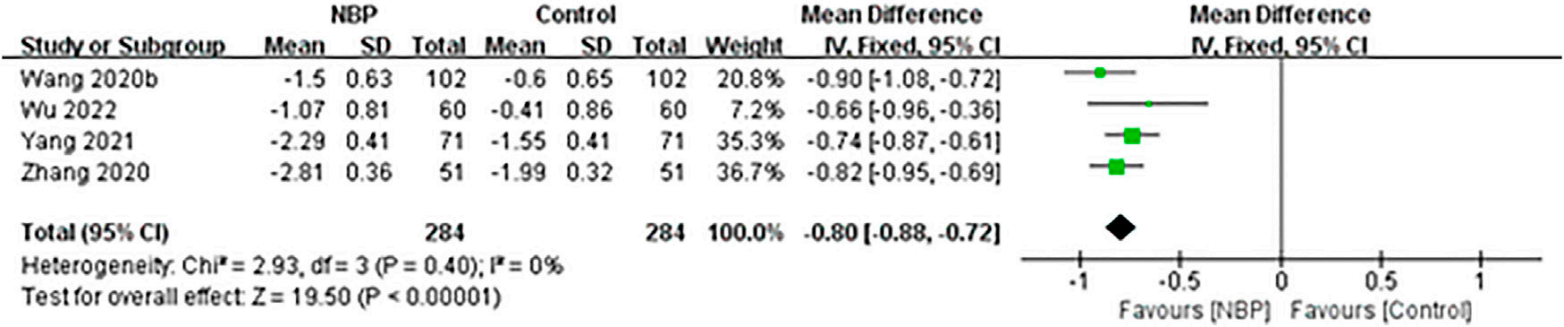
Meta-analysis of the modified Rankin Scale after treatment, as an index of functional independence.

The remaining four trials (Wang, 2020; Zhang et al., 2020; Yang et al., 2021; Wu, 2022) reported mRS scores as means and standard deviations (SDs), which could not be converted to dependency rates. Across these four trials, fixed-effect meta-analysis showed a significant decrease in the mRS score among patients receiving NBP (MD -0.80, 95% CI -0.88 to -0.72; participants = 568; studies = 4; I^2^ = 0%; Figure 5).

#### BI

Twenty-three trials used the BI to assess the performance of personal basic activities of daily living after NBP treatment (Zhou et al., 2015; Zheng et al., 2016; Fu et al., 2017; Gao et al., 2017; Liu et al., 2018a; Zhang et al., 2018a; Lin et al., 2018; Xu, 2018; Zhang and Li, 2018; Qin and Han, 2019; Chen et al., 2021; Chen and Qian, 2021; Ye et al., 2021; Zhang et al., 2021; Si et al., 2022; Wu, 2022; Zhu, 2022) at follow-up of either 1 month (Fu, 2015) or 3 months (Li, 2017a; Li et al., 2017; Lv et al., 2018; Ma and Xiao, 2018; Yang et al., 2021). One trial (Zheng et al., 2016) could not be included in the meta-analysis because it did not report BI scores after treatment.

**FIGURE 6:**
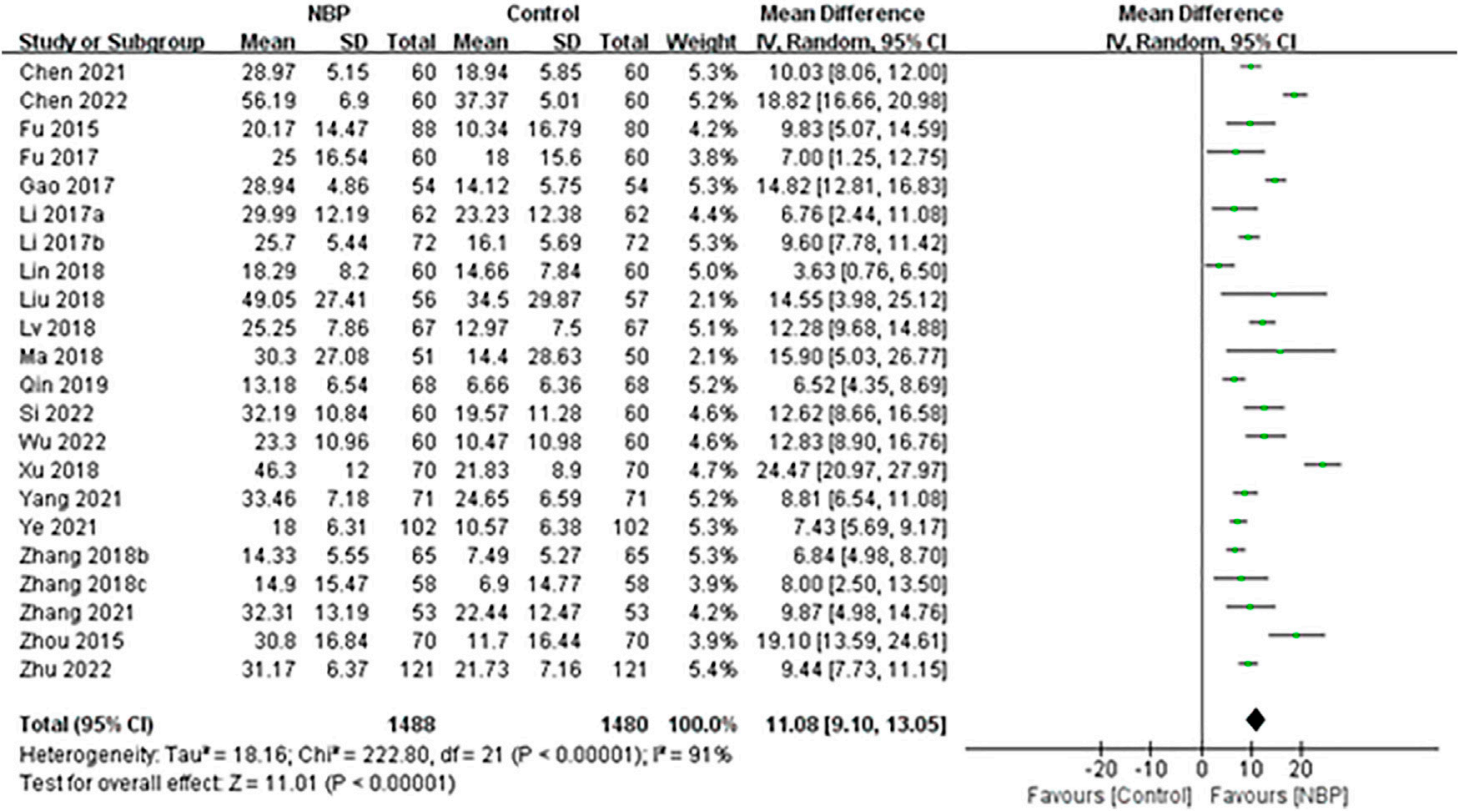
Meta-analysis of Barthel Index at the end of treatment or follow-up, as an index of basic activities of daily living.

The remaining 22 trials, involving 2,975 participants (Fu, 2015; Zhou et al., 2015; Li, 2017a; Fu et al., 2017; Gao et al., 2017; Li et al., 2017; Liu et al., 2018a; Zhang et al., 2018a; Lin et al., 2018; Lv et al., 2018; Ma and Xiao, 2018; Xu, 2018; Zhang and Li, 2018; Qin and Han, 2019; Chen et al., 2021; Chen and Qian, 2021; Yang et al., 2021; Ye et al., 2021; Zhang et al., 2021; Si et al., 2022; Wu, 2022; Zhu, 2022) of whom 7 (0.24%) were lost to follow-up, reported BI as means and SDs, which could not be converted to dependency rates. Random-effect meta-analysis showed that BI increased significantly more among patients receiving NBP (MD 11.08, 95% CI 9.10 to 13.05; participants = 2,968; studies = 22; I^2^ = 91%; Figure 6).

### Global neurological impairment improvement

#### NIHSS

Forty-eight trials used the NIHSS to assess neurological deficit, but two of them did not report specific NIHSS scores (Ma and Xiao, 2018; Wang et al., 2020b). The remaining 46 trials reported NIHSS scores at baseline and at the end of treatment (Lv, 2015; Yan and Ma, 2015; Zhou et al., 2015; Dong et al., 2016; Wang and Li, 2016; Xu et al., 2016; Zheng et al., 2016; Li, 2017a; Li, 2017b; Gao et al., 2017; Li et al., 2017; Liu et al., 2018a; Zhang et al., 2018a; Zhang et al., 2018b; Chang and Ma, 2018; Lin et al., 2018; Lv et al., 2018; Xiong and Hong, 2018; Xu, 2018; Yu, 2018; Zhang, 2018; Zhang and Li, 2018; Chen et al., 2019; Jin, 2019; Qin and Han, 2019; Wu, 2019; Li et al., 2020a; Wang et al., 2020a; Wang, 2020; Zhang et al., 2020; Liu et al., 2021a; Zhu et al., 2021a; Zhu et al., 2021b; Chen et al., 2021; Chen and Qian, 2021; Li, 2021; Pang et al., 2021; Ye et al., 2021; Si et al., 2022; Wu, 2022; Zhu, 2022) and follow-up (Fu, 2015; Fu et al., 2017; Chang, 2018; Bai, 2019; Pan et al., 2019; Zhou et al., 2020). These trials involved 7,316 participants, of whom 7 (0.10%) died and 26 (0.36%) were lost to follow-up.

**FIGURE 7:**
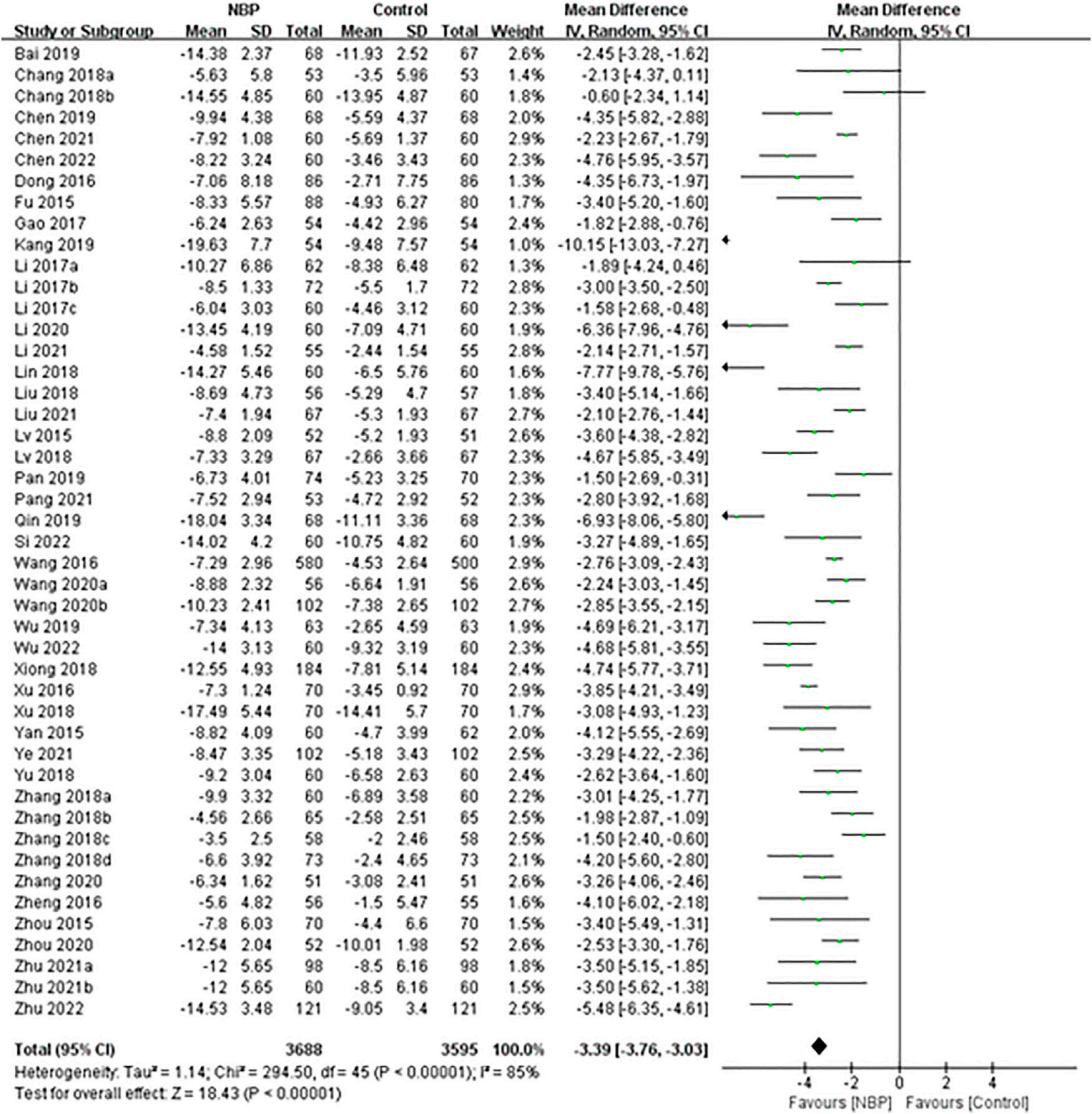
Meta-analysis of NIHSS score at the end of treatment or follow-up, as an index of neurological deficit.

Random-effect meta-analysis of these 46 trials showed a significant decrease in the NIHSS score among patients receiving NBP (MD -3.39, 95% CI -3.76 to -3.03; participants = 7,283; studies = 46; I^2^ = 85%; Figure 7).

#### CSS

Four trials (Cui et al., 2005a; Cui et al., 2005b; Wei et al., 2012; Li, 2018) with 543 participants assessed neurological deficit using the CSS score (Cui et al., 2005a; Cui et al., 2005b; Wei et al., 2012; Li, 2018). Random-effect meta-analysis showed that NBP significantly decreased CSS score (MD -4.16, 95% CI -7.60 to -0.73; participants = 543; studies = 4; I^2^ = 91%; Supplementary Figure S2).

#### Other scales

Two trials assessed neurological deficit using the Cerebrovascular Disease Rehabilitation Medical Plans (Xu et al., 2006) and Assessment Standard Scale or the modified Edinburgh-Scandinavia Stroke Scale (MESSS) (Zhang et al., 2021). A third trial (Fu et al., 2017) used an unidentified scale. Meta-analysis of all three trials showed that NBP was associated with significantly greater improvement of neurological function than the control intervention (MD -3.73, 95% CI -4.64 to -2.82; participants = 345; studies = 3; I^2^ = 0%; Supplementary Figure S3).

### Adverse events

Thirty-one trials (Cui et al., 2005a; Cui et al., 2005b; Xu et al., 2006; Zhou et al., 2015; Wang and Li, 2016; Zheng et al., 2016; Li, 2017a; Gao et al., 2017; Zhang et al., 2018a; Chang, 2018; Ma and Xiao, 2018; Xiong and Hong, 2018; Yu, 2018; Zhang and Li, 2018; Bai, 2019; Jiang, 2019; Jin, 2019; Qin and Han, 2019; Wu, 2019; Li et al., 2020a; Wang, 2020; Zhang et al., 2020; Zhou et al., 2020; Liu et al., 2021a; Li, 2021; Pang et al., 2021; Ye et al., 2021; Zhang et al., 2021; Si et al., 2022; Wu, 2022; Zhu, 2022) reported that adverse events occurred, three of which (Wu, 2019; Li et al., 2020a; Zhu, 2022) reported that adverse events occurred without providing details. The most frequent adverse events reported by the other 28 trials were elevated transaminase, rash and gastrointestinal discomfort.

Ten trials (Cui et al., 2005a; Cui et al., 2005b; Xu et al., 2006; Zhou et al., 2015; Zheng et al., 2016; Zhang et al., 2018a; Jiang, 2019; Wang, 2020; Zhang et al., 2020; Si et al., 2022) reported elevated transaminase, but five trials (Zhang et al., 2018a; Jiang, 2019; Wang, 2020; Zhang et al., 2020; Si et al., 2022) did not clearly describe the specific change in transaminase. The other five trials reported an increase in alanine transaminase in 1.39-17.53% of participants who received NBP, compared to 5-5.88% of controls (Cui et al., 2005a; Cui et al., 2005b; Xu et al., 2006; Zhou et al., 2015; Zheng et al., 2016). Two of those five trials (Cui et al., 2005b; Xu et al., 2006) also observed that 1.69-6.19% of NBP participants and 0-2.94% of controls had elevated aspartate aminotransferase. Meta-analysis of the five trials indicated significantly higher incidence of elevated alanine transaminase in the intervention group than in the control group (RR 2.63, 95% CI 1.34 to 5.14; participants = 713; studies = 5; I^2^ = 0%, Supplementary Figure S4). In contrast, meta-analysis of the two trials reporting aspartate aminotransferase findings found that the incidence of aspartate aminotransferase abnormality did not differ significantly between the two groups (RR 2.24, 95% CI 0.64 to 7.79; participants = 318; studies = 2; I^2^ = 0%, Supplementary Figure S5).

Eleven trials (Zheng et al., 2016; Li, 2017a; Chang, 2018; Ma and Xiao, 2018; Yu, 2018; Zhang and Li, 2018; Qin and Han, 2019; Wang, 2020; Liu et al., 2021a; Li, 2021; Wu, 2022) involving 1,396 participants reported rash in 0-1.96% of participants in the intervention group and 0-8.33% in the control group. Meta-analysis showed no significant difference in the incidence of rash between the two groups (RR 0.66, 95% CI 0.32 to 1.37; participants = 1,396; studies = 11; I^2^ = 0%; Supplementary Figure S6).

Seventeen trials (Cui et al., 2005b; Xu et al., 2006; Zhou et al., 2015; Wang and Li, 2016; Li, 2017a; Zhang et al., 2018a; Chang, 2018; Xiong and Hong, 2018; Zhang and Li, 2018; Jiang, 2019; Jin, 2019; Qin and Han, 2019; Wang, 2020; Zhou et al., 2020; Ye et al., 2021; Zhang et al., 2021; Si et al., 2022) reported that 1.09-6.15% of the intervention group and 0-13.2% of controls experienced abdominal and gastrointestinal symptoms, such as loss of appetite, nausea, and vomiting. In addition, three trials (Chang, 2018; Zhang and Li, 2018; Zhang et al., 2021) reported mild gastrointestinal bleeding in 0-3.45% of participants in the intervention group and 1.67-5.66% in the control group.

Seven trials (Gao et al., 2017; Chang, 2018; Yu, 2018; Zhang and Li, 2018; Liu et al., 2021a; Li, 2021; Ye et al., 2021) reported gingival bleeding in 0-15.52% of participants in the intervention group and 0.98-18.97% in the control group. Three trials (Gao et al., 2017; Bai, 2019; Pang et al., 2021) reported hemorrhagic transformation after cerebral infarction in 1.47-3.7% of participants in the intervention group and 3.7-11.54% in controls. Three trials (Jin, 2019; Zhou et al., 2020; Ye et al., 2021) reported that dizziness in 0.98-3.85% of the intervention group and 0-1.85% of controls.

Infrequent adverse events in the intervention group included mild hallucination in one case (Cui et al., 2005b) (1.0%), agitation in one case (Xu et al., 2006) (1.7%), sleepiness in two cases (Jin, 2019) (3.70%), headache in one case (Zheng et al., 2016) (1.79%), transient chest tightness in two cases (Ma and Xiao, 2018; Liu et al., 2021a) (1.49-1.96%), hypotension in two cases (Zhang et al., 2020) (3.92%), diarrhea in one case (Jiang, 2019) (1.96%), fatigue in one case (Zhou et al., 2020) (1.92%), and subcutaneous bleeding in three cases (Gao et al., 2017) (5.6%).

### Assessment of reporting bias

**FIGURE 8:**
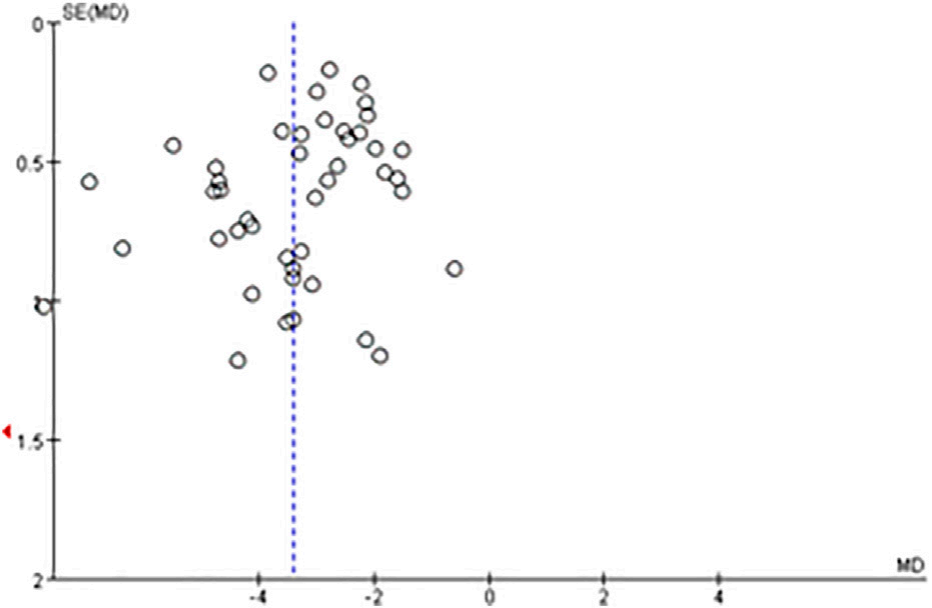
Funnel plot for evaluating publication bias in the meta-analysis of NIHSS score at the end of treatment or follow-up (46 trials).

The meta-analysis of changes of neurological deficit based on NIHSS score involved the greatest number of studies (46), so this meta-analysis was analyzed by funnel plot. The plot appeared symmetrical, suggesting no significant publication bias (Figure 8).

### Subgroup analysis and sensitivity analysis

Subgroup analyses had been conducted to determine whether there were differences in treatment effects among different forms of NBP. The subgroup analyses were not performed for composite outcome of death and dependency because there were fewer than ten trials available. Among the remaining outcomes, the treatment effects were similar for most subgroups, except for death (Supplementary Figures S8–10). Using soft capsules appeared to be associated with a significant reduction in death, while either injections or sequential therapy showed no similar reduction (Supplementary Figure S7). We had also planned to perform sensitivity analyses to explore the influence of study quality on effect size by excluding trials whose overall risk of bias was “high” or “some concern”. However, we did not perform sensitivity analysis because all trials but one (Cui et al., 2005b) were categorized as being at “high” or “some concern” of overall risk of bias.

## Discussion

This updated systematic review, which includes 54 RCTs not examined in a 2010 systematic review, confirms earlier conclusions that NBP mitigates neurological deficit, improves daily living after acute ischemic stroke, and is generally well tolerated. Unfortunately, there were too few trials and their quality was too low to conclude whether NBP reduces risk of long-term death or dependency after ischemic stroke. Nevertheless, we can conclude that NBP reduces risk of short-term death after ischemic stroke. Even though we were able to include many additional RCTs, despite excluding trials with up to100 patients, most of the trials in our review were of lower quality and were considered at high overall risk of bias. Our analysis highlights the need for NBP trials that analyze long-term mortality and disability, particularly using the mRS.

Stroke is highly disabling and can lead to severe neurological impairment in the acute phase. Studies suggest that 4%-38% of acute stroke patients present with a reduced level of consciousness or coma, 13%-48% with confusion or delirium, and 37%-78% with dysphagia on admission (Li et al., 2016; Powers et al., 2019). It may be difficult to administer drugs orally to such patients, who are at increased risk of aspiration pneumonia or airway obstruction (Hannawi et al., 2013). Especially for such patients, NBP soft capsules have been reformulated as an injection. The present review, unlike the previous 2010 review, included NBP formulated as an injection. Our review also included sequential NBP therapy (Jin et al., 2020), in which NBP is initially given as an injection, and then later as a soft capsule after patients stabilize or are discharged. Our results are consistent with a randomized, double-blind, double-dummy trial involving 573 patients with ischemic stroke that showed that sequential NBP treatment lasting 90 days effectively decreased mRS scores and improved functional outcomes (Mamtilahun et al., 2013).

The adverse events reported in 31 of the 57 studies in our review did not include any serious events; the most frequent events were elevated transaminase, rash and gastrointestinal discomfort. Similarly, a phase IV, multicenter, prospective, open-label trial showed that NBP injection was safe and associated with an overall adverse event rate of 3.28% in patients with acute ischemic stroke (Li et al., 2019a). Even though NBP injections have been used in the clinic for more than a decade, the evidence base still needs to examine adverse events over much longer periods.

Mechanistic studies of NBP indicate that it can accelerate microcirculatory blood flow, dilate microvascular caliber, improve cerebral circulation, protect mitochondrial function, improve mitochondrial energy pump, enhance the oxidative stress response of the nervous system, inhibit neuronal apoptosis and autophagy, reduce infarct size, and improve energy metabolism after cerebral ischemia (Wang et al., 2010). NBP downregulates AQP4 and matrix metalloprotease-9 (Mamtilahun et al., 2021), which may help explain how it protects the blood-brain barrier in the acute phase of ischemic stroke (Hu et al., 2014; Feng et al., 2018; Li et al., 2019b). NBP can promote collateriogenesis, neurogenesis, and angiogenesis; increase axonal growth; and strengthen white matter integrity (Yang et al., 2015; Sun et al., 2017; Zhou et al., 2019; Wang et al., 2020c; Liu et al., 2021b; Qu et al., 2021; Wei et al., 2021). NBP can accelerate the recovery of cerebral blood flow and reduce cognitive impairment (Xiong et al., 2017; Li et al., 2019c). It can inhibit platelet activation via inhibition of cPLA2-mediated TXA2 synthesis and platelet phosphodiesterase (Ye et al., 2015). NBP can also attenuate ischemia reperfusion brain injury by suppressing inflammation, promoting remyelination, inhibiting neuronal apoptosis, and increasing regional blood flow (Wen et al., 2016; Qin et al., 2019; Yang et al., 2019; Li et al., 2020b). These pleiotropic effects make NBP a powerful weapon against acute ischemic stroke and ensuing injury.

Although we included only RCTs that clearly described the method of patient allocation and that included more than 100 patients, most trials in our review proved to be at high risk of bias in the domains of placebo use, allocation concealment, and blinding of efficacy evaluation (One study (Cui et al., 2005b) was a notable exception). These issues are common among clinical trials involving cerebrovascular diseases in China (He et al., 2012). Thus, these methodological aspects should be improved in order to increase RCT quality (Liu et al., 2018b). Concerted efforts are needed from government policymakers, clinical research organizations, international clinical trial monitoring agencies, and clinical training programs in order to bolster clinical trial quality in China.

## Limitations

This systematic review has several limitations. First, the methodological quality of the eligible studies is generally low. Most studies did not report key information about randomization or blinding, or complete outcomes data, which, to a certain extent, affects the reliability of the results. Although we did not impose geographic constraints during our literature search, all included studies were conducted in China, raising the question of generalizability to other populations. Second, assessment scales and endpoint definitions varied across studies, contributing to heterogeneity.

## Conclusion

The results of our study suggest that DL-3-n-butylphthalide reduces the rate of short-term death and improves the degree of neurological deficit in patients with acute ischemic stroke, while showing a good safety profile. More research is needed to assess efficacy at reducing long-term death and disability.

## Data Availability

The original contributions presented in the study are included in the article/Supplementary Material, further inquiries can be directed to the corresponding author.

## References

[B1] Acute Ischemic Stroke Diagnosis and Treatment Guidelines Writing Group Cerebrovascular Diseases Group Neurology Branch of Chinese Medical Association (2010). Guidelines for diagnosis and treatment of acute ischemic stroke in China 2010. Chin. J. Neurology 43 (2), 16–19. Google Scholar

[B2] Author Anonymous (1996). The criteria of the 4th congress of Chinese cerebrovascular diseases. Chin. J. Neurol. 29 (6), 379–380. Google Scholar

[B3] Bai H. (2019). Effect of butylphthalide injection combined with rt-PA intravenous thrombolysis on NIHSS score and quality of daily life in patients with acute cerebral infarction. China Minkang Med. 31 (9), 20–22. Google Scholar

[B4] Chang B. Ma L. (2018). Effect of butylphthalide on plasma lysophosphatidic acid level in patients with acute watershed infarction and its short-term efficacy analysis. China Mod. Med. 25 (19), 73–76. Google Scholar

[B5] Chang Y. (2018). Clinical observation of butylphthalide injection combined with alteplase in the treatment of acute cerebral infarction[J]. North. Pharm. 15 (10), 28–29. Google Scholar

[B6] Chen L. Wu C. Xie Z. Sun C. Zhou S. (2021). Size-dependent nonlinear optical properties of Gd_2_O_2_S:Tb^3+^ scintillators and their doped gel glasses. Molecules 20 (5), 85–87. 10.3390/molecules27010085 10.3390/molecules27010085 | Google Scholar PMC8746479 35011317

[B7] Chen P. Zhang X. Liu Z. (2019). Clinical effect of butylphthalide combined with edaravone on acute ischemic stroke and its effect on apoptosis. Chin. J. Pract. Nerv. Dis. 22 (15), 1643–1648. Google Scholar

[B8] Chen S. Qian M. (2021). Effect of butylphthalide combined with atorvastatin calcium in the treatment of older adult patients with acute cerebral infraction and its influence on neurological function and oxidative stress. Chin. J. Prim. Med. Pharm. 28 (2), 267–271. Google Scholar

[B9] Chinese Medical Association Cerebrovascular Diseases Group Neurology Branch of Chinese Medical Association (2015). Guidelines for diagnosis and treatment of acute ischemic stroke in China 2014. Chin. J. Neurology 48 (4), 246–257. Google Scholar

[B10] Chinese Society of Neurology (2018). Chinese Stroke Society. Chinese guidelines for diagnosis and treatment of acute ischemic stroke 2018. Chin. J. Neurology 51 (9), 666–682. Google Scholar

[B11] Cui L. Li S. Lu F. (2005). The multicentric randomized study of dl-3-butylphthalide in the treatment of acute moderate ischemic. Chin. J. Cerebrovasc. Dis. 2 (3), 112–115. Google Scholar

[B12] Cui L. Liu X. Zhu Y. (2005). Effects of dl-3-butylphthalide on treatment of acute ischemic stroke with moderate symptoms: A multi-center, randomized, double-blind, placebo-controll trial. Chin. J. Neurol. 38 (4), 251. Google Scholar

[B13] Dong T. Niu X. Liu L. (2016). Clinical observation of probucol combined with butylphthalide in the treatment of patients with acute cerebral infarction. China Pharm. 27 (26), 3655–3658. Google Scholar

[B14] Feng L. Sharma A. Niu F. Huang Y. Lafuente J. V. Muresanu D. F. (2018). TiO_2_-Nanowired delivery of DL-3-n-butylphthalide (DL-NBP) attenuates blood-brain barrier disruption, brain edema formation, and neuronal damages following concussive head injury. Mol. Neurobiol. 55 (1), 350–358. 10.1007/s12035-017-0746-5 PubMed Abstract | 10.1007/s12035-017-0746-5 | Google Scholar 28856586

[B15] Fu D. Nong W. Yu S. (2017). Observation on the therapeutic effect of butylphthalide combined with ginkgo damo on acute cerebral infarction. J. Front. Med. 7 (11), 94–96. Google Scholar

[B16] Fu Z. (2015). The clinical study of the treatment for acute cerebral infarction super early with Dl-3n-butylphthalide and sodium injection [D]. Google Scholar

[B17] Gao J. Yang Z. Ma Y. (2017). Efficacy observation of alteplase combined with butylphthalide in the treatment of acute ischemic stroke. Chin. Pharm. 20 (10), 1817–1819. Google Scholar

[B18] Hannawi Y. Hannawi B. Rao C. P. Suarez J. I. Bershad E. M. (2013). Stroke-associated pneumonia: Major advances and obstacles. Cerebrovasc. Dis. 35 (5), 430–443. 10.1159/000350199 PubMed Abstract | 10.1159/000350199 | Google Scholar 23735757

[B19] Hatano S. (1976). Experience from a multicentre stroke register: A preliminary report. Bull. World Health Organ. 54 (5), 541–553. PubMed Abstract | Google Scholar 1088404 PMC2366492

[B20] He S. Wu S. Zeng Q. Zhang S. Lin S. Zhang C. (2012). Assessment of methodological quality and outcome measures of acute stroke randomized controlled trials in China in recent 15 years. J. Evid. Based. Med. 5 (3), 174–182. 10.1111/j.1756-5391.2012.01190.x PubMed Abstract | 10.1111/j.1756-5391.2012.01190.x | Google Scholar 23672224

[B21] Higgins J. P. T. Sterne J. A. C. Savović J. (2016). Revised Cochrane risk of bias tool for randomized trials (RoB 2.0). UK: cochrane collaboration. Google Scholar

[B22] Hu J. Wen Q. Wu Y. Li B. Gao P. (2014). The effect of butylphthalide on the brain edema, blood-brain barrier of rats after focal cerebral infarction and the expression of Rho A. Cell biochem. Biophys. 69 (2), 363–368. 10.1007/s12013-013-9808-0 PubMed Abstract | 10.1007/s12013-013-9808-0 | Google Scholar 24442989

[B23] Jiang J. (2019). Effect of butylphthalide combined with edaravone on inflammatory factors in patients with acute cerebral infarction. Henan Med. Res. 29 (11), 2024–2025. Google Scholar

[B24] Jin H. Zhou H. Shao J. (2020). Effect of sequential treatment of butylphthalide on neurological function and cognitive function in patients with acute cerebral infarction. Chin. Med. Clin. 20 (5), 737–739. Google Scholar

[B25] Jin S. (2019). Clinical effect of butylphthalide injection combined with rosuvastatin calcium tablets in the treatment of acute progressive cerebral infarction. Clin. Res. Pract. 4 (30), 42–46. 10.7619/jcmp.201917013 10.7619/jcmp.201917013 | Google Scholar

[B26] Kleindorfer D. O. Towfighi A. Chaturvedi S. Cockroft K. M. Gutierrez J. Lombardi-Hill D. (2021). 2021 guideline for the prevention of stroke in patients with stroke and transient ischemic attack: A guideline from the American heart association/American stroke association. Stroke 52 (7), e364–e467. 10.1161/STR.0000000000000375 PubMed Abstract | 10.1161/STR.0000000000000375 | Google Scholar 34024117

[B27] Li C. Wang L. Yu L. (2020). Effect of butylphthalide combined with edaravone on neurological function and vascular endothelial function inf patients with acute cerebral infarction. Renowned Dr. 11 (2), 290–291. Google Scholar

[B28] Li J. Liu Y. Zhang X. Chen R. Zhang L. Xue J. (2019). Dl-3-N-Butylphthalide alleviates the blood-brain barrier permeability of focal cerebral ischemia reperfusion in mice. Neuroscience 10 (413), 99–107. 10.1016/j.neuroscience.2019.06.020 10.1016/j.neuroscience.2019.06.020 | Google Scholar 31247236

[B29] Li J. Wang D. Tao W. Dong W. Zhang J. Yang J. (2016). Early consciousness disorder in acute ischemic stroke: Incidence, risk factors and outcome. BMC Neurol. 16 (1), 140. 10.1186/s12883-016-0666-4 PubMed Abstract | 10.1186/s12883-016-0666-4 | Google Scholar 27535026 PMC4989496

[B30] Li M. Meng N. Guo X. Niu X. Zhao Z. Wang W. (2020). DL-3-n-Butylphthalide promotes remyelination and suppresses inflammation by regulating AMPK/SIRT1 and STAT3/NF-κB signaling in chronic cerebral hypoperfusion. Front. Aging Neurosci. 9 (12), 137. 10.3389/fnagi.2020.00137 PubMed Abstract | 10.3389/fnagi.2020.00137 | Google Scholar PMC7296049 32581761

[B31] Li Q. Tian W. Chen L. Wang C. Can Y. Jin X. (2017). Effect of Dl-3-n-butylphthalide injection on vascular endothelial growth factor and tumor necrosis factor α in serum of patients with acute cerebral infarction. Med. Recapitulate 23 (5), 1001–1005. Google Scholar

[B32] Li S. Wang Y. Zheng H. (2019). Safety and efficacy of administration of dl-3-n-butylphthalide for acute ischemic stroke: A phase IV, multicenter, prospective, open-lable trial. Chin. J. Stroke 14 (5), 450–455. Google Scholar

[B33] Li W. Wei D. Lin J. Liang J. Xie X. Song K. (2019). DL-3-n-Butylphthalide reduces cognitive impairment induced by chronic cerebral hypoperfusion through GDNF/GFRα1/Ret signaling preventing hippocampal neuron apoptosis. Front. Cell. Neurosci. 13 (13), 351. 10.3389/fncel.2019.00351 PubMed Abstract | 10.3389/fncel.2019.00351 | Google Scholar 31456664 PMC6701226

[B34] Li X. (2017). Clinical efficacy of butylphthalide in the treatment of acute cerebral infarction and its effect on serum C-reactive protein. Chin. J. Prim. Med. Pharm. 24 (8), 1194–1197. Google Scholar

[B35] Li X. (2021). Clinical evaluation of Danhong injection combined with butylphthalide injection in the treatment of acute cerebral infarction. China Prac. Med. 16 (1), 137–139. Google Scholar

[B36] Li Y. (2017). Effects of butylphthalide combined with atorvastatin treatment on neurologic impairment and hemorheology in patients with acute cerebral infarction. Nerve Inj. Funct. Reconstr. 12 (1), 22–24. Google Scholar

[B37] Li Y. D. (2018). Efficacy of butylphthalide combined with edaravone in the treatment of acute ischemic stroke and its effect on inflammatory factors and neurological function. Mod. Pract. Med. 30 (3), 361–363. Google Scholar

[B38] Lin C. Min J. Pan D. (2018). Efficacy analysis of butylphthalide soft capsules combined with atorvastatin calcium tablets in the treatment of cerebral infarction. Strait Pharm. 30 (8), 119–120. Google Scholar

[B39] Liu H. Yan J. En H. (2021). Effect of butyphthal combined with alteplase on neurological function and coagulation function and serological indexes as TNF-α hs-CRP and hcy in patients with acute ischemic stroke. Hebei Med. 27 (1), 160–156. Google Scholar

[B40] Liu M. Zhang S. Zhu Y. (2018). Consensus on clinical research norms of acute stroke in China 2018. Chin. J. Neurology 51 (4), 247–255. Google Scholar

[B41] Liu X. Liu R. Fu D. Wu H. Zhao X. Sun Y. (2021). DL-3-n-butylphthalide inhibits neuroinflammation by stimulating foxp3 and Ki-67 in an ischemic stroke model. Aging (Albany NY) 13 (3), 3763–3778. 10.18632/aging.202338 PubMed Abstract | 10.18632/aging.202338 | Google Scholar 33461169 PMC7906154

[B42] Liu X. Xin W. Cao Q. (2018). Effect of butylphthalide on collateral circulation in patients with acute cerebral infarction. Med. J. Natl. Defending Forces Southwest China 28 (2), 23–26. Google Scholar

[B43] Lv F. (2015). Efficacy of fluvastatin combined with butylphthalide soft capsule in the treatment of acute cerebral infarction and its effect on serum IL-6. Chin. J. Gerontology 35 (18), 5154–5155. Google Scholar

[B44] Lv X. Peng J. Zeren Z. (2018). Effect of early intervention in lower limb repetitive training combined with SBR on motor function and rehabilitation of patients with acute cerebral infarction. J. Hunan Norm. Univ. Med. Sci. 15 (4), 167–170. Google Scholar

[B45] Lyden P. D. (2021). Cerebroprotection for acute ischemic stroke: Looking ahead. Stroke 52 (9), 3033–3044. 10.1161/STROKEAHA.121.032241 PubMed Abstract | 10.1161/STROKEAHA.121.032241 | Google Scholar 34289710 PMC8384682

[B46] Ma X. Xiao Y. (2018). Efficacy and safety of butylphthalide injection in the treatment of progressive stroke. China Minkang Med. 30 (12), 65–66. Google Scholar

[B47] Mamtilahun M. Cui L. Zhu Y. Gao S. Peng B. Ni J. (2013). Ninety-day administration of DL-3-n-butylphthalide for acute ischemic stroke: A randomized, double-blind trial. Chin. Med. J. 126 (18), 3405–3410. PubMed Abstract | Google Scholar 24034079

[B48] Mamtilahun M. Wei Z. Qin C. Wang Y. Tang Y. Shen F. X. (2021). DL-3n-Butylphthalide improves blood-brain barrier integrity in rat after middle cerebral artery occlusion. Front. Cell. Neurosci. 12 (14), 610714. 10.3389/fncel.2020.610714 10.3389/fncel.2020.610714 | Google Scholar PMC7835508 33510620

[B49] Moher D. Liberati A. Tetzlaff J. Altman D. G. PRISMA Group (2009). Preferred reporting items for systematic reviews and meta-analyses: The PRISMA statement. BMJ 21 (339), b2535. 10.1136/bmj.b2535 PubMed Abstract | 10.1136/bmj.b2535 | Google Scholar PMC2714657 19622551

[B50] Pan W. Wang M. Yuan Y. (2019). Clinical observation on butylphthalide used within 24 hours of intravenous thrombolysis in treating acute ischemic cerebral infarction. Acad. J. Shanghai Univ. Traditional Chin. Med. 33 (5), 12–16. Google Scholar

[B51] Pang X. Hao X. Guo H. (2021). Clinical efficacy and effect of butylphthalide combined with low-dose alteplase on neuroendocrine factors of patients with acute cerebral infarction. Prog. Mod. Biomed. 21 (3), 562–566. Google Scholar

[B52] Powers W. J. Rabinstein A. A. Ackerson T. Adeoye O. M. Bambakidis N. C. Becker K. (2019). Guidelines for the early management of patients with acute ischemic stroke: 2019 update to the 2018 guidelines for the early management of acute ischemic stroke: A guideline for healthcare professionals from the American heart association/American stroke association. Stroke 50 (12), e344–e418. 10.1161/STR.0000000000000211 PubMed Abstract | 10.1161/STR.0000000000000211 | Google Scholar 31662037

[B53] Qin C. Zhou P. Wang L. Mamtilahun M. Li W. Zhang Z. (2019). DL-3-N-butylphthalide attenuates ischemic reperfusion injury by improving the function of cerebral artery and circulation. J. Cereb. Blood Flow. Metab. 39 (10), 2011–2021. 10.1177/0271678X18776833 PubMed Abstract | 10.1177/0271678X18776833 | Google Scholar 29762050 PMC6775578

[B54] Qin N. Han F. (2019). Efficacy of sequential therapy of butylphthalide injection and capsule in adjuvant treatment of APCI and its effects on neurological function, hemorheology and inflammatory factors. Laboratory Med. Clin. 16 (2), 115–117. Google Scholar

[B55] Qu M. Zhao J. Zhao Y. Sun J. Liu L. Wei L. (2021). Vascular protection and regenerative effects of intranasal DL-3-N-butylphthalide treatment after ischaemic stroke in mice. Stroke Vasc. Neurol. 6 (1), 74–79. 10.1136/svn-2020-000364 PubMed Abstract | 10.1136/svn-2020-000364 | Google Scholar 32958696 PMC8005898

[B56] Si X. Xue H. Liu B. (2022). Effect of argatroban with butylphthalide on patients with ischemic stroke. Chin. J. Pract. Med. 49 (1), 105–108. Google Scholar

[B57] Sulter G. Steen C. De Keyser J. (1999). Use of the barthel index and modified rankin scale in acute stroke trials. Stroke 30 (8), 1538–1541. 10.1161/01.str.30.8.1538 PubMed Abstract | 10.1161/01.str.30.8.1538 | Google Scholar 10436097

[B58] Sun Y. Cheng X. Wang H. Mu X. Liang Y. Luo Y. (2017). DL-3-n-butylphthalide promotes neuroplasticity and motor recovery in stroke rats. Behav. Brain Res. 30 (329), 67–74. 10.1016/j.bbr.2017.04.039 PubMed Abstract | 10.1016/j.bbr.2017.04.039 | Google Scholar 28442357

[B59] Wang C. (2020). Clinical effects of Butylphthalide combined with Argatroban in treatment of patients with acute cerebral infarction. Med. J. Chin. People's Health 32 (16), 19–21. Google Scholar

[B60] Wang D. Liu M. Wu B. Hao Z. L. Li J. He S. (2010). Dl-3-butylphthalide for acute ischemic stroke: A systematic review. Chin. J. Evid-based Med. 10 (2), 189–195. Google Scholar

[B61] Wang H. Li R. (2016). Clinical observation on the treatment of 1080 cases of acute cerebral infarction with Butylphthalide soft capsule. Med. Inf. 29 (17), 44–45. Google Scholar

[B62] Wang H. Zhang T. Huang J. Sun X. J. (2013). 3-N-butylphthalide (NBP) attenuates the amyloid-β-induced inflammatory responses in cultured astrocytes via the nuclear factor-κB signaling pathway. Cell. Physiol. biochem. 32 (1), 235–242. 10.1159/000350139 PubMed Abstract | 10.1159/000350139 | Google Scholar 23899885

[B63] Wang M. Feng Y. Yuan Y. Gui L. Wang J. Gao P. (2020). Use of l-3-n-Butylphthalide within 24 h after intravenous thrombolysis for acute cerebral infarction. Complement. Ther. Med. 52, 102442. 10.1016/j.ctim.2020.102442 PubMed Abstract | 10.1016/j.ctim.2020.102442 | Google Scholar 32951710

[B64] Wang W. Wang X. (2004). The criteria of the 6th congress of Chinese cerebrovascular diseases. Chin. J. Neurol. 37 (4), 346. Google Scholar

[B65] Wang Y. Han B. Hong S. (2020). Effect of butylphthalide on acute cerebral lnfarction and caveolin-1. Drugs Clin. Pract. 20, 90–92. Google Scholar

[B66] Wang Y. Li Z. Zhao X. Wang D. Li H. Xian Y. (2017). Stroke care quality in China: Substantial improvement, and a huge challenge and opportunity. Int. J. Stroke 12 (3), 229–235. 10.1177/1747493017694392 PubMed Abstract | 10.1177/1747493017694392 | Google Scholar 28381200

[B67] Wang Y. Shen Y. Liu Z. Gu J. Xu C. Qian S. (2020). DL-NBP (DL-3-N-butylphthalide) treatment promotes neurological functional recovery accompanied by the upregulation of white matter integrity and HIF-1α/VEGF/Notch/Dll4 expression. Front. Pharmacol. 24 (10), 1595. 10.3389/fphar.2019.01595 PubMed Abstract | 10.3389/fphar.2019.01595 | Google Scholar PMC6993069 32038259

[B68] Wei N. Wei Y. Li B. (2012). The effects of butylphthalide on homocysteine and C-reactive protein levels in patients with acute cerebral infarction. Chin. J. Postgraduates Med. 35 (4), 8–10. Google Scholar

[B69] Wei Z. Chen D. Lee M. J. H. Zhao Y. Gu X. Yu S. P. (2021). DL-3-n-butylphthalide increases collateriogenesis and functional recovery after focal ischemic stroke in mice. Aging Dis. 12 (7), 1835–1849. 10.14336/AD.2020.1226 PubMed Abstract | 10.14336/AD.2020.1226 | Google Scholar 34631224 PMC8460296

[B70] Wen X. Tang M. Qi D. Huang X. J. Liu H. Z. Zhang F. (2016). Butylphthalide suppresses neuronal cells apoptosis and inhibits JNK-caspase3 signaling pathway after brain ischemia /reperfusion in rats. Cell. Mol. Neurobiol. 36 (7), 1087–1095. 10.1007/s10571-015-0302-7 PubMed Abstract | 10.1007/s10571-015-0302-7 | Google Scholar 27015680 PMC11482424

[B71] Wu A. (2022). Effect of butylphthalide on neurological function in elderly patients with acute cerebral infarction. Mod. Med. Health Res. 6 (2), 38–41. Google Scholar

[B72] Wu S. Wu B. Liu M. Chen Z. Wang W. Anderson C. S. (2019). Stroke in China: Advances and challenges in epidemiology, prevention, and management. Lancet. Neurol. 18 (4), 394–405. 10.1016/S1474-4422(18)30500-3 PubMed Abstract | 10.1016/S1474-4422(18)30500-3 | Google Scholar 30878104

[B73] Wu W. (2019). Effect of butylphthalide sodium chloride injection on neurological function and vascular endothelial function in patients with acute ischemic stroke. Huaihai Med. 37 (2), 195–197. Google Scholar

[B74] Xiong L. Hong Y. (2018). Clinical efficacy and safety observation of butylphthalide in the treatment of acute cerebral infarction. J. Anhui Health Vocat. Tech. Coll. 17 (4), 43–44. Google Scholar

[B75] Xiong Z. Lu W. Zhu L. Zeng L. Shi C. Jing Z. (2017). DL-3-n-Butylphthalide treatment enhances hemodynamics and ameliorates memory deficits in rats with chronic cerebral hypoperfusion. Front. Aging Neurosci. 26 (9), 238. 10.3389/fnagi.2017.00238 PubMed Abstract | 10.3389/fnagi.2017.00238 | Google Scholar PMC5526838 28798681

[B76] Xu C. (2018). Effect of butylphthalide soft capsule combined with atorvastatin calcium tablets on collateral circulation establishment of cerebral infarction. Modern diagnosis and treatment 29 (19), 32–34. Google Scholar

[B77] Xu C. Xu J. Zang W. Song Y. Li X. Li Y. (2006). Evaluation of butylphthalide in treating acute ischemic stroke. Chin. J. New Drugs Clin. Remedies 25 (7), 508–511. Google Scholar

[B78] Xu Y. Liu Y. Zhao X. (2016). Effects of butylphthalide injection on acute cerebral infarction and on serum sTRAIL, OPG and TNF-α. Chin. J. Pract. Nerv. Dis. 19 (13), 3–4. Google Scholar

[B79] Yan Y. Ma J. (2015). Impact of butylphthalide on homocysteine, CRP and nerve function in patients with acute progressive cerebral infarction. Chin. Pharm. 2015 (11), 1911–1913. Google Scholar

[B80] Yang C. Guo A. Li Y. Shi K. Shi F. D. Li M. (2019). DL-3-n-butylphthalide reduces neurovascular inflammation and ischemic brain injury in mice. Aging Dis. 10 (5), 964–976. 10.14336/AD.2019.0608 PubMed Abstract | 10.14336/AD.2019.0608 | Google Scholar 31595195 PMC6764730

[B81] Yang L. Li J. Xu S. Cai J. Lei H. Liu D. M. (2015). L-3-n-butylphthalide promotes neurogenesis and neuroplasticity in cerebral ischemic rats. CNS Neurosci. Ther. 21 (9), 733–741. 10.1111/cns.12438 PubMed Abstract | 10.1111/cns.12438 | Google Scholar 26215907 PMC6493029

[B82] Yang R. Li Y. Yan S. (2021). Evaluation of curative effect of hutylphthalide sodium chloride injection combined with rt-PA for broadened time window thrombolysis in treatment of patients with acute cerebral infarction. Chin. J. TCM WM Crit. Care 28 (3), 316–319. Google Scholar

[B83] Ye J. Ling Q. Peng F. (2021). Influence of Butylphthalide combined with dual antiplatelet therapy on patients with acute cerebral infarction. China J. Mod. Med. 31 (9), 60–66. Google Scholar

[B84] Ye J. Zhao L. Zhang S. Zhang Y. Chen L. Hu L. (2015). DL-3-n-butylphthalide inhibits platelet activation via inhibition of cPLA2-mediated TXA2 synthesis and phosphodiesterase. Platelets 26 (8), 736–744. 10.3109/09537104.2014.989826 PubMed Abstract | 10.3109/09537104.2014.989826 | Google Scholar 25734213

[B85] Yu F. (2018). Treatment of acute cerebral infarction with butylphthalide injection combined with Danhong injection in 60 patients. Chin. J. New Drugs 27 (24), 57–60. Google Scholar

[B86] Zhang J. (2018). Effect of butylphthalide injection on serum basic fibroblast growth factor and placental-derived growth factor in patients with acute cerebral infarction. China Pract. Med. J. 45 (14), 122–124. Google Scholar

[B87] Zhang L. Li B. (2018). Impact of rt-PA combined with butyphthalide on acute ischemic stroke patients. Pract. J. Cardiac Cereb. Pneumal Vasc. Dis. 26 (8), 118–121. Google Scholar

[B88] Zhang M. Wu T. Yang J. Li X. Cheng Z. Yu X. (2020). 3D visualization ablation planning system assisted microwave ablation for hepatocellular carcinoma (diameter >3): A precise clinical application. BMC Cancer 31 (1), 44–47. 10.1186/s12885-020-6519-y PubMed Abstract | 10.1186/s12885-020-6519-y | Google Scholar PMC6972027 31959147

[B89] Zhang R. Xu Y. Gao L. (2018). Therapeutic effect and safety evaluation of dual antiplatelet combined with butylphthalide sequential therapy for acute cerebral infarction. China Foreign Med. Treat. 37 (26), 132–134. Google Scholar

[B90] Zhang Y. Lu R. Nie F. Guo Z. Zeng X. (2018). Clinical observation of butylphthalide and sodium chloride injection in the treatment of patients with acute cerebral infarction. Chin. J. Pract. Nerv. Dis. 21 (4), 420–423. Google Scholar

[B91] Zhang Y. Xu J. Han X. (2021). Clinical study of alteplase combined with butylphthalide in the treatment of acute cerebral infarction. Chin. J. Ration. Drug Use 18 (11), 67–70. Google Scholar

[B92] Zhao W. Wu C. Dornbos D. 3rd Li S. Song H. Wang Y. (2020). Multiphase adjuvant neuroprotection: A novel paradigm for improving acute ischemic stroke outcomes. Brain Circ. 186 (1), 11–18. 10.4103/bc.bc_58_19 PubMed Abstract | 10.4103/bc.bc_58_19 | Google Scholar PMC7045534 32166195

[B93] Zheng Z. Chen Y. Cheng Q. (2016). Efficacy of sequential treatment with dl-3-n-butylphthalide for posterior circulation infarction. J. Qiqihar Univ. Med. 37 (22), 2769–2771. Google Scholar

[B94] Zhou J. Duan L. Zheng Z. (2015). Efficacy of butylphthalide sodium chloride injection plus Edaravone for acute cerebral infarction. Eval. Analysis Drug Use Chin. Hosp. 15 (4), 37–39. Google Scholar

[B95] Zhou P. Wang L. Qu M. Shen H. Zheng H. R. Deng L. D. (2019). DL-3-N-butylphthalide promotes angiogenesis and upregulates sonic hedgehog expression after cerebral ischemia in rats. CNS Neurosci. Ther. 25 (6), 748–758. 10.1111/cns.13104 PubMed Abstract | 10.1111/cns.13104 | Google Scholar 30784219 PMC6515698

[B96] Zhou X. Xue Y. Guo J. (2020). Influence of butyphthalide sequential therapy combined with dual anti-platelet therapy on serum levels of HMGB1, MMP-9, 3-MST, Fibulin-5 in patients with ACI. J. Brain Nerv. Dis. 28 (8), 498–503. Google Scholar

[B97] Zhu D. (2022). Clinical efficacy of butylphthalide combined with edaravone in patients with acute cerebral infarction. Chin. J. Mod. Drug Appl. 16 (3), 123–126. Google Scholar

[B98] Zhu K. Li H. Chang J. (2021). Effect of butylphthalide combined with breviscapine on neurological function and cognitive function in patients with acute cerebral infarction. J. Hubei Minzu University·Medical Ed. 38 (2), 57–63. Google Scholar

[B99] Zhu K. Li H. Chang J. (2021). Effect of early dual antiplatelet drugs combined with butylphthalide in the treatment of acute ischemic stroke. Heilongjiang Med. Pharm. 44 (4), 172–174. Google Scholar

