# Supplementary material for: DL-3-n-butylphthalide for acute ischemic stroke: An updated systematic review and meta-analysis of randomized controlled trials
Source: Front Pharmacol. 2022 Sep 2;13:963118. doi: 10.3389/fphar.2022.963118 (PMC9479342; doi:10.3389/fphar.2022.963118)
Supplement: Supplementary file 1 [file DataSheet1.docx]

**Supplementary Table S1. Search process used for Ovid Medline.**



| 1. Cerebrovascular disorders/  2. exp Brain ischemia/  3. exp Cerebrovascular accident/  4. exp Hypoxia-ischemia, brain/  5. (stroke$ or apoplex$ or cerebral vasc$ or cerebrovasc$ or cva).tw.  6. (brain or cerebr$ or cerebell$ or vertebrobasil$ or hemispher$ or intracran$ or intracerebral or infratentorial or supratentorial or middle cerebr$ or mca$ or anterior circulation).tw.  7. (isch?emi$ or infarct$ or thrombo$ or emboli$ or occlus$ or hypoxi$).tw.  8. 6 and 7  9. 1 or 2 or 3 or 4 or 5 or 8  10. (Butylphthalide$ or 3-n-butylphthalide$ or l-3-n-Butylphthalide$ or DL-3-n-Butylphthalid- e$ or NBP$ or BuPh$ or dinbente$ or L-NBP$).tw  11. 9 and 10  12. limit 11 to humans |
| --- |

**Supplementary Table S2.** Baseline characteristics of included RCTs.

| Study | Participant characteristics | | | | Methodology | | | Interventions | | Neurolog-ical deficit score | Baseline comparison | Adverse events |
| --- | --- | --- | --- | --- | --- | --- | --- | --- | --- | --- | --- | --- |
|  | Age  NBP group/control group  (age) | | Number of cases  (male) | Onset time | Randomiz-ation methods | Allocation concealment | Blinding | NBP group | Control group |  |  |  |
| Fu 2015 ^[21]^ | 38-78 | 35-73 | 168 (93) | < 48h | Random number | No description | Not mentioned | NBP+  other | Other | NIHSS | Comparable | Not reported |
| Yu 2018 ^[22]^ | 62.05± 9.45 | 61.28±7.55 | 121 (81) | < 14d | Random number | No description | Blinding of participants | NBP+  other | Other | NIHSS | Comparable | Yes |
| Liu 2018 ^[23]^ | - | | 120 | < 48h | Random number | No description | Not mentioned | NBP+  other | Other | NIHSS | Comparable | Not reported |
| Lv 2018 ^[24]^ | 37-78 | 35-80 | 134 (72) | < 72h | Random number | No description | Not mentioned | NBP+  other | Other | NIHSS | Comparable | Not reported |
| Lv 2015 ^[25]^ | 37-72 | 35-73 | 103 (62) | 6-24h | Random number | No description | Not mentioned | NBP+  other | Other | NIHSS | Comparable | Not reported |
| Wu 2019 ^[26]^ | 39-78 | 42-76 | 126 (69) | ≤ 72h | Random number | No description | Not mentioned | NBP+  other | Other | NIHSS | Comparable | Yes |
| Zhou 2015 ^[27]^ | 46-73 | | 140 (84) | < 24h | Random number | No description | Not mentioned | NBP+  other | Other | NIHSS | Comparable | Yes |
| Cui 2005a ^[18]^ | 40-75 | | 144 | < 72h | Random number | Yes | Blinding of study personnel | NBP+  other | Placebo +other | CSS | Comparable | Yes |
| Cui 2005b ^[19]^ | 40-75 | | 201 | < 72h | Random number | Yes | Blinding of participants and study personnel | NBP+  other | Placebo +other | CSS | Comparable | Yes |
| Chang 2018a ^[28]^ | 52-86 | 51-88 | 106 (62) | < 48h | Random number | No description | Not mentioned | NBP+  other | Other | NIHSS | Comparable | Not reported |
| Chang 2018b ^[29]^ | 36-73 | 37-74 | 120 (63) | ≤ 4.5h | Random number | No description | Not mentioned | NBP+  other | Other | NIHSS | Comparable | Yes |
| Zhang 2018a ^[30]^ | 45-70 | 46-70 | 120 (68) | < 72h | Random number | No description | Not mentioned | NBP+  other | Other | NIHSS | Comparable | Not found |
| Zhang 2018b ^[31]^ | 63.64±6.55 | 65.31±7.56 | 130 (76) | < 72h | Random number | No description | Not mentioned | NBP+  other | Other | NIHSS | Comparable | Yes |
| Zhang 2018c ^[32]^ | 60-76 | 60-75 | 116 (62) | < 6h | Random number | No description | Not mentioned | NBP+  other | Other | NIHSS | Comparable | Yes |
| Zhang 2018d ^[33]^ | 38-79 | 36-76 | 146 (81) | ≤ 72h | Random number | No description | Not mentioned | NBP+  other | Other | NIHSS | Comparable | Not reported |
| Xu 2006 ^[34]^ | 35-70 | | 119 (78) | 6-72h | Random number | No description | Not mentioned | NBP+  other | Other | Rehabilitat-ion medical plan and evaluation standard | Comparable | Yes |
| Li 2017a ^[35]^ | 43-76 | 48-80 | 124 (80) | < 24h | Computer  random number | Yes | Blinding of participants and study personnel | NBP+  other | Other | NIHSS | Comparable | Yes |
| Li 2017b ^[36]^ | 36-80 | | 144 (88) | < 24h | Random number | No description | Not mentioned | NBP+  other | Other | NIHSS | Comparable | Not found |
| Li 2017c ^[37]^ | 39-85 | 36-86 | 120 (79) | 6-72h | Random number | No description | Not mentioned | NBP+  other | Other | NIHSS | Comparable | Not reported |
| Li 2018d ^[38]^ | 45-78 | 46-79 | 108 (69) | ≤ 72h | Random number | No description | Not mentioned | NBP+  other | Other | CSS | Comparable | Not reported |
| Lin 2018 ^[39]^ | 40-73 | 39-75 | 120 (63) | < 48h | Random number | No description | Not mentioned | NBP+  other | Other | NIHSS | Comparable | Not reported |
| Xiong 2018 ^[40]^ | 52-73 | 51-74 | 368 (235) | ≤ 48h | Random number | No description | Not mentioned | NBP+  other | Other | NIHSS | Comparable | Yes |
| Wang 2016 ^[20]^ | 56-78 | 55-79 | 1080 (592) | < 7d | Random number | No description | Not mentioned | NBP+  other | Placebo +other | NIHSS | Comparable | Yes |
| Bai 2019 ^[41]^ | 46-68 | 48-67 | 135 (73) | < 4h | Random number | No description | Not mentioned | NBP+  other | Other | NIHSS | Comparable | Yes |
| Qin 2019 ^[42]^ | 40-75 | 40-74 | 136 (75) | < 72h | Random number | No description | Not mentioned | NBP+  other | Other | NIHSS | Comparable | Yes |
| Fu 2017 ^[43]^ | 45-85 | | 120 (74) | < 48h | Random number | No description | Not mentioned | NBP+  other | Other | Not clear | Comparable | No |
| Dong 2016 ^[44]^ | 61.90±10.75 | 61.85±10.62 | 172 (101) | ≤ 72h | Random number | No description | Not mentioned | NBP+  other | Other | NIHSS | Comparable | Not found |
| Xu 2016 ^[45]^ | 51-75 | 50-75 | 140 (73) | < 24h | Random number | No description | Not mentioned | NBP+  other | Other | NIHSS | Comparable | Not reported |
| Xu 2018 ^[46]^ | 52-62 | 51-63 | 140 (79) | < 48h | Random number | No description | Not mentioned | NBP+  other | Other | NIHSS | Comparable | Not reported |
| Zheng 2016 ^[47]^ | 43-80 | | 120 (75) | < 48h | Random number | No description | Not mentioned | NBP+  other | Other | NIHSS | Comparable | Yes |
| Chen 2019 ^[48]^ | 38-79 | | 136 (71) | 6-72h | Random number | No description | Not mentioned | NBP+  other | Other | NIHSS | Comparable | Not reported |
| Wei 2012 ^[49]^ | 45-73 | 46-75 | 110 (66) | < 48h | Random number | No description | Not mentioned | NBP+  other | Other | CSS | Comparable | Not reported |
| Yan 2015 ^[50]^ | 40-80 | | 122 (70) | 6-72h | Random number | No description | Not mentioned | NBP+  other | Other | NIHSS | Comparable | Not found |
| Ma 2018 ^[51]^ | 42-83 | | 101 (60) | ≤ 72h | Random number | No description | Not mentioned | NBP+  other | Other | No data available | Comparable | Yes |
| Gao 2017 ^[52]^ | 63.94±4.85 | 65.81±5.36 | 108 (52) | ≤ 4.5h | Random number | No description | Not mentioned | NBP+  other | Other | NIHSS | Comparable | Yes |
| Kang 2019 ^[53]^ | 70.62±11.58 | 71.03±10.39 | 108 (52) | ＜ 72h | Random number | No description | Not mentioned | NBP+  other | Other | NIHSS | Comparable | Yes |
| Pan 2019 ^[54]^ | 52.31±14.26 | 51.92±16.35 | 144 (75) | ＜ 4.5h | Random number | No description | Not mentioned | NBP+  other | Other | NIHSS | Comparable | Not found |
| Jiang 2020 ^[55]^ | 59.86±4.17 | 60.03±4.20 | 102 (53) | < 72h | Random number | No description | Not mentioned | NBP+  other | Other | Not reported | Comparable | Yes |
| Li 2020 ^[56]^ | 62.12±6.45 | 62.25±6.56 | 120 (66) | < 72h | Random number | No description | Not mentioned | NBP+  other | Other | NIHSS | Comparable | Yes |
| Wang 2020a ^[57]^ | 54.7±6.5 | 52.8±9.6 | 112 (54) | < 72h | Random number | No description | Not mentioned | NBP+  other | Other | NIHSS | Comparable | Not found |
| Wang 2020b ^[58]^ | 56.34±8.16 | 56.84±8.42 | 204 (121) | ≤ 33h | Random number | No description | Not mentioned | NBP+  other | Other | NIHSS | Comparable | Yes |
| Zhang 2020 ^[59]^ | 64.12±4.64 | 63.20±5.43 | 102 (74) | < 24h | Random number | No description | Not mentioned | NBP+  other | Other | NIHSS | Comparable | Yes |
| Zhou 2020 ^[60]^ | 58.98±6.94 | 60.12±6.65 | 104 (50) | 6-72h | Random number | No description | Not mentioned | NBP+  other | Other | NIHSS | Comparable | Yes |
| Chen 2021 ^[61]^ | 68.76±3.19 | 67.96±3.42 | 120 (75) | < 48h | Random number | No description | Not mentioned | NBP+  other | Other | NIHSS | Comparable | Not reported |
| Li 2021 ^[62]^ | 58.11±4.97 | 58.01±5.01 | 110 (64) | ≤ 24h | Random number | No description | Not mentioned | NBP+  other | Other | NIHSS | Comparable | Yes |
| Liu 2021 ^[63]^ | 59.00±11.80 | 58.30±12.40 | 134 (71) | ≤ 6h | Random number | No description | Not mentioned | NBP+  other | Other | NIHSS | Comparable | Yes |
| Pang 2021 ^[64]^ | 58.14±7.38 | 57.30±6.22 | 105 (58) | < 4.5h | Random number | No description | Not mentioned | NBP+  other | Other | NIHSS | Comparable | Yes |
| Yang 2021 ^[65]^ | 50.15±5.27 | 50.21±5.13 | 142 (73) | 4.5-24h | Random number | No description | Not mentioned | NBP+  other | Other | Not reported | Comparable | Not found |
| Ye 2021 ^[66]^ | 56.97±6.62 | 55.43±6.17 | 204 (116) | < 24h | Random number | No description | Not mentioned | NBP+  other | Other | NIHSS | Comparable | Yes |
| Zhang 2021 ^[67]^ | 59.42±15.63 | 59.71±16.24 | 106 (55) | < 3h | Random number | No description | Not mentioned | NBP+  other | Other | modified Edinburgh-Scandinavia stroke scale | Comparable | Yes |
| Zhu 2021a ^[68]^ | 63.12±5.12 | 63.61±5.35 | 196 (115) | < 12h | Random number | No description | Not mentioned | NBP+  other | Other | NIHSS | Comparable | Not reported |
| Zhu 2021b ^[69]^ | 65.12±4.12 | 65.61±4.35 | 120 (61) | < 6h | Random number | No description | Not mentioned | NBP+  other | Other | NIHSS | Comparable | Not reported |
| Chen 2022 ^[70]^ | 63.02±4.96 | 62.85±4.76 | 120 (77) | ≤ 48h | Random number | No description | Not mentioned | NBP+  other | Other | NIHSS | Comparable | Not reported |
| Si 2022 ^[71]^ | 62.44±6.76 | 63.22±7.02 | 120 (76) | < 72h | Random number | No description | Not mentioned | NBP+  other | Other | NIHSS | Comparable | Yes |
| Wu 2022 ^[72]^ | 78.32±5.21 | 78.31±5.26 | 120 (73) | < 48h | Random number | No description | Not mentioned | NBP+  other | Other | NIHSS | Comparable | Yes |
| Zhu 2022 ^[73]^ | 56.8±4.6 | 57.1±4.2 | 242 (132) | < 24h | Random number | No description | Not mentioned | NBP+  other | Other | NIHSS | Comparable | Yes |
| Wang 2020c ^[74]^ | 51.77±15.63 | 52.69±15.84 | 178 (105) | < 4.5h | Random number | No description | Not mentioned | NBP+  other | Other | No data available | Comparable | Not reported |

**Supplementary Figure S1.** Assessment of risk of bias in included trials.


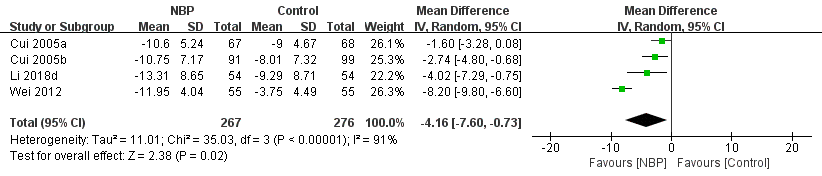


**Supplementary Figure S2.** Meta-analysis of CSS score at the end of treatment, as an index of neurological deficit.


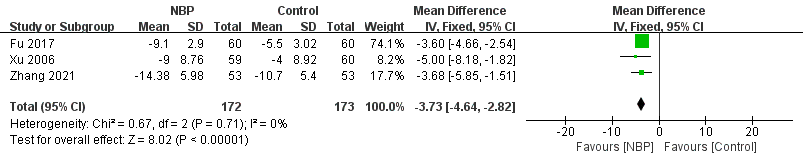


**Supplementary Figure S3.** Meta-analysis of other scores at the end of treatment or follow-up, as indices of neurological deficit.


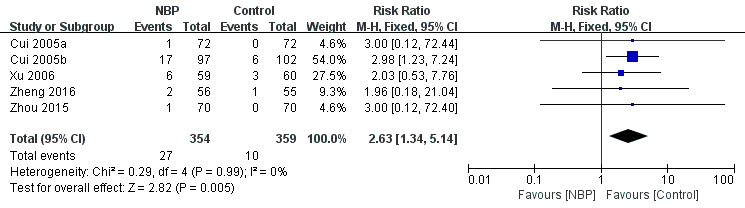


**Supplementary Figure S4.** Meta-analysis of the incidence of abnormal alanine transaminase.


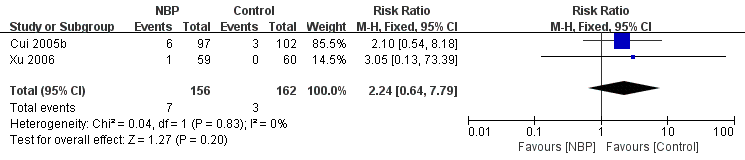


**Supplementary Figure S5.** Meta-analysis of the incidence of abnormal aspartate aminotransferase.


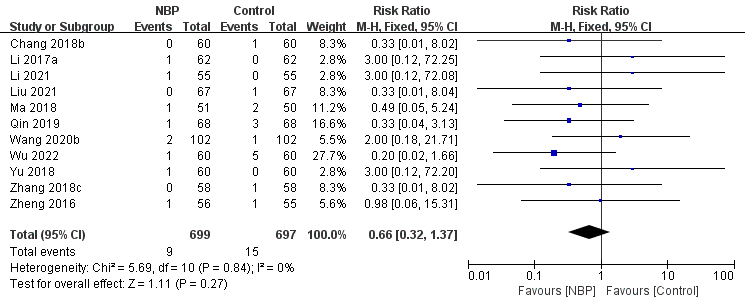


**Supplementary Figure S6.** Meta-analysis of the incidence of rash.


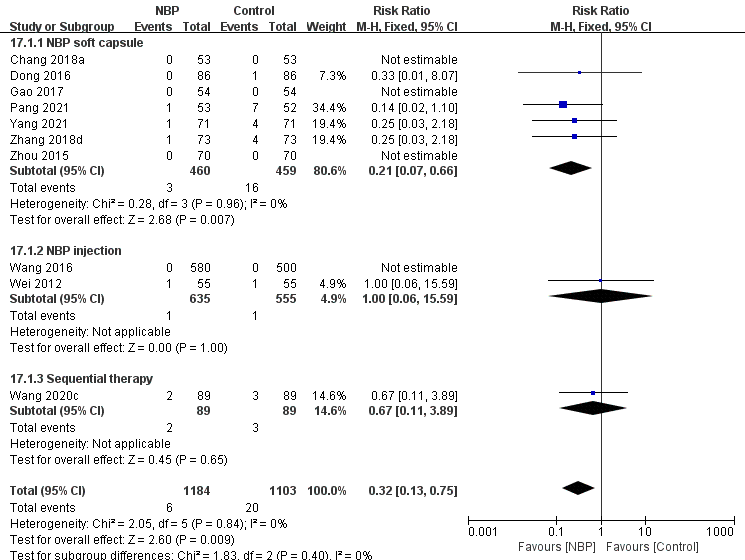


**Supplementary Figure S7.** Meta-analysis of death during the treatment period or follow-up (NBP type subgroup).


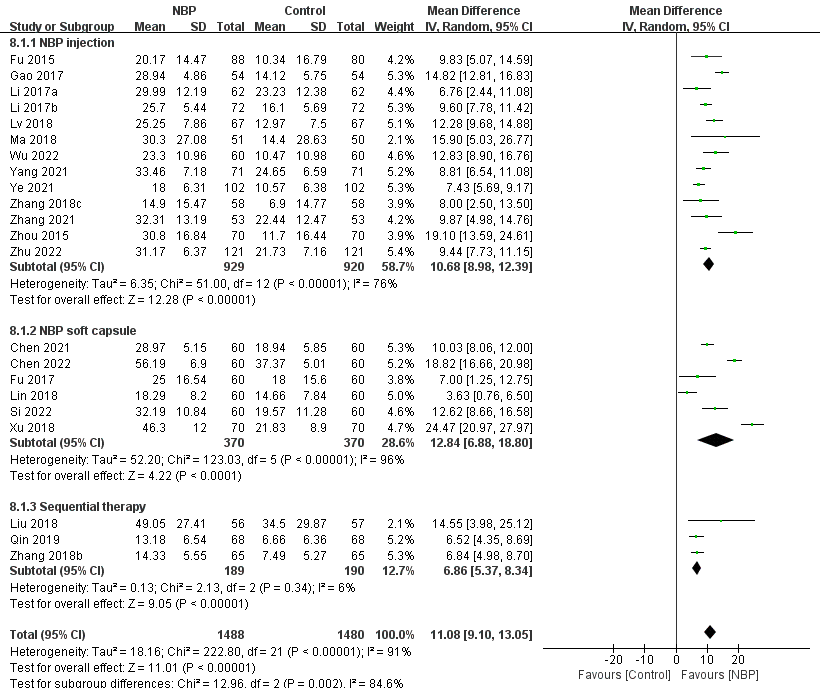


**Supplementary Figure S8.** Meta-analysis of Barthel Index at the end of treatment or follow-up, as an index of basic activities of daily living (NBP type subgroup).


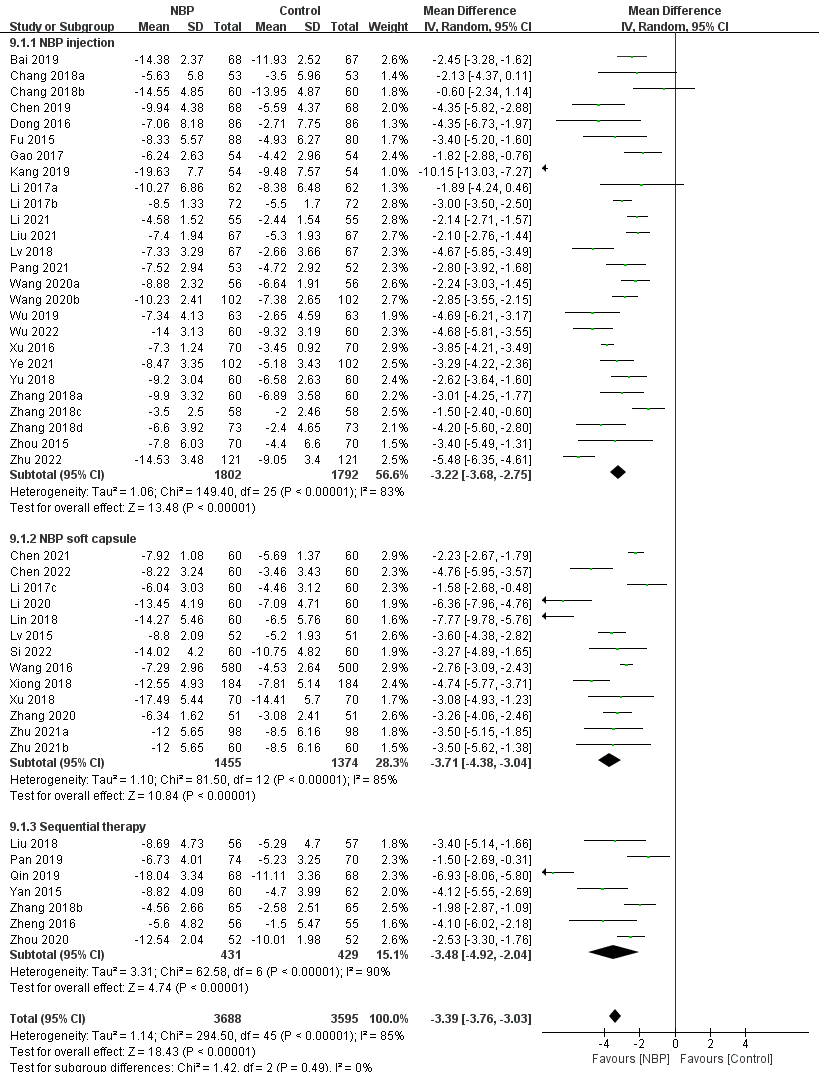


**Supplementary Figure S9.** Meta-analysis of NIHSS score at the end of treatment or follow-up, as an index of neurological deficit (NBP type subgroup).


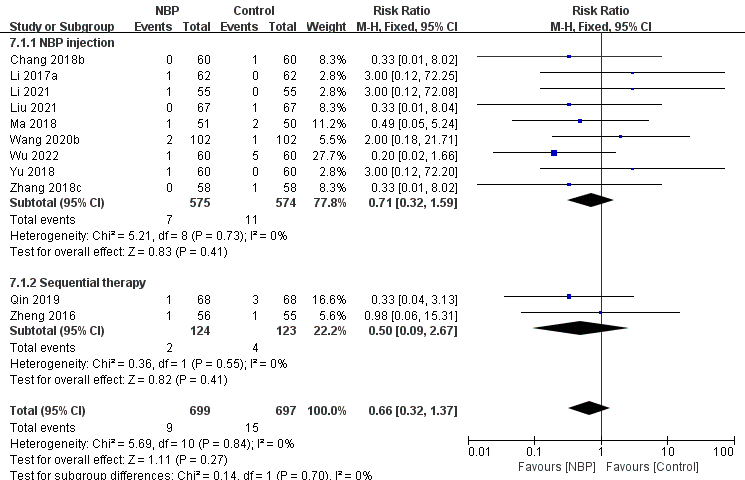


**Supplementary Figure S10.** Meta-analysis of the incidence of rash (NBP type subgroup).

.
